# Cancer Cell Metastasis: From Molecular Mechanisms to Therapeutic Strategies

**DOI:** 10.1002/mco2.71026

**Published:** 2026-10-02

**Authors:** Yupeng Zhao, Wenchao Xu, Yibo Wang, Haoran Huang, Yu Su, Li Zhou, Jianzhou Liu, Junchao Guo

**Affiliations:** ^1^ Department of General Surgery Peking Union Medical College Hospital Chinese Academy of Medical Sciences and Peking Union Medical College Beijing China; ^2^ Key Laboratory of Research in Pancreatic Tumor Chinese Academy of Medical Sciences Beijing China; ^3^ National Infrastructures for Translational Medicine Peking Union Medical College Hospital Beijing China; ^4^ State Key Laboratory of Complex Severe and Rare Diseases Peking Union Medical College Hospital Chinese Academy of Medical Sciences and Peking Union Medical College Beijing China; ^5^ Institute of Clinical Medicine, Peking Union Medical College Hospital Chinese Academy of Medical Sciences and Peking Union Medical College Beijing China

**Keywords:** metastasis, metastatic tumor cells, migratory tumor microenvironment, targeted therapy

## Abstract

Cancer metastasis is a spatiotemporal dynamic process jointly driven by the intrinsic evolution of cancer cells and continuous microenvironmental selection, rather than a simple linear accumulation of genetic alterations. The metastatic diversity of cancers is driven by heterogeneity in physicochemical properties, cellular composition, and signaling networks among primary tumors, dissemination routes, and target organs. Currently, the stage‐dependent and route‐dependent molecular mechanisms underlying metastasis and potential pan‐cancer therapeutic targets remain incompletely defined. Here, we systematically discuss the genomic, chromosomal, and epigenetic evolution of metastatic tumor cells (MTCs) following their spatiotemporal trajectory from in situ evolution to metastatic colonization. We describe their migration behaviors and interaction patterns within the migratory tumor microenvironment across hematogenous, lymphatic, and perineural routes, and the establishment and remodeling of organ‐specific metastatic niches. We also review the progress and limitations of metastasis‐oriented diagnostic stratification and therapeutic interventions. We argue for the shift of antimetastatic strategies from target‐centric approaches to precise interventions that match timing, routes, biomarkers, and endpoints. By integrating the molecular mechanisms underlying spatiotemporal changes during cancer metastasis, this review delineates the spatiotemporal evolutionary landscape of metastasis and the MTC–microenvironment interaction patterns, thereby offering insights for future clinical translation of metastasis‑targeted therapies.

## Graphical abstract

The schematic representation of intrinsic and extrinsic influences of cancer cell metastasis. Genomic evolution of metastatic tumor cells (MTCs) promotes their metastatic phenotypes and reconstruct the primary tumor microenvironment. During MTCs migration along different routes (blood circulation, lymphatic vessels, and perineuronal spaces), the migratory microenvironment actively interacts with MTCs and reprograms the local tumor microenvironment to permit MTCs migration, adhesion, extravasation, and colonization at distant organs such as liver, lung, and brain. Targeted therapy and immunotherapies aimed at molecular and cellular components, or intermediates governing such interactions shows great potentials in metastasis intervention.

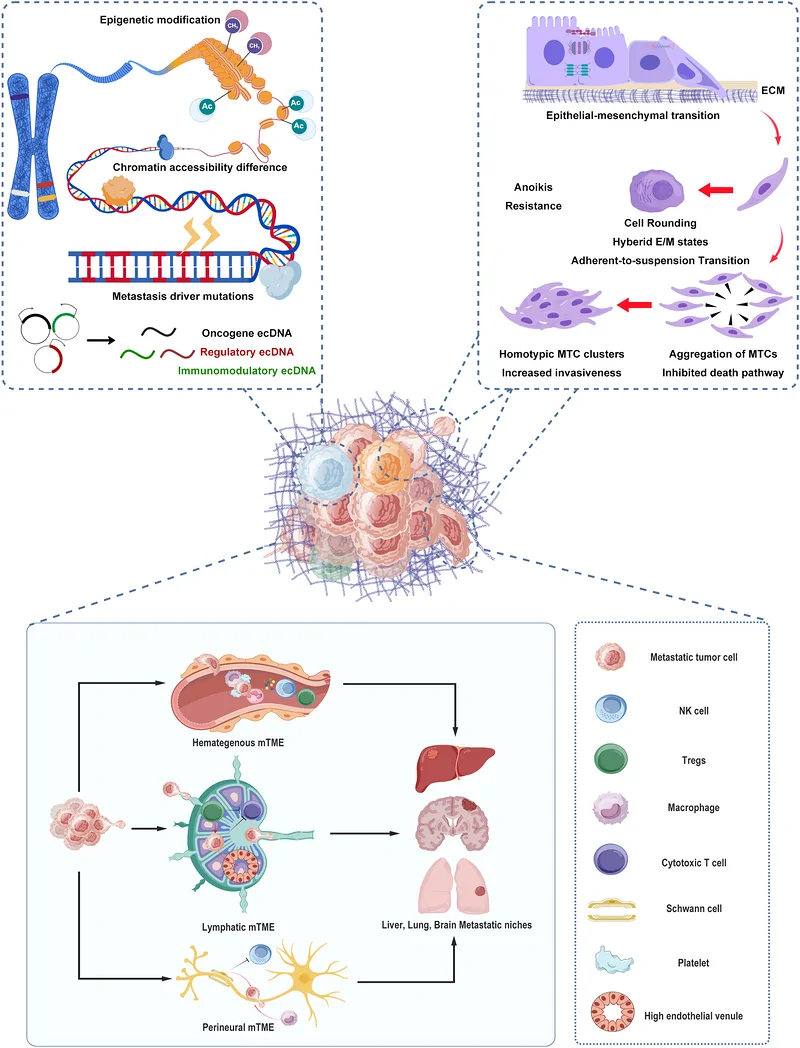

## 1 Introduction

Metastasis is a defining event in malignant progression that is closely associated with treatment failure and patient mortality [1]. Rather than a simple spatial relocation of cancer cells, metastasis is now understood as a multistep cascade encompassing local invasion, intravasation, tissue transit, extravasation, dormancy, and metastatic outgrowth, ultimately resulting in invasive growth in distant organs [2, 3, 4]. Throughout this process, metastatic tumor cells (MTCs) must not only possess intrinsic metastatic competence but also withstand multiple selective pressures, including loss of adhesion, mechanical injury, oxidative and metabolic stress, immune surveillance, and organ‐specific barriers. Moreover, MTCs are exposed to diverse microenvironment throughout the metastatic process, and the physicochemical and biological differences among primary sites, dissemination routes, and target organs constitute major sources of metastatic heterogeneity. Successful distant seeding requires cancer cells to overcome these successive constraints. Accordingly, reciprocal interactions between cancer cells and local niches are integral to the metastatic cascade and ultimately determine metastatic fate.

Conceptual models of metastasis have evolved from anatomical theories of dissemination to ecological and evolutionary frameworks. In 1889, Stephen Paget proposed the “seed and soil” hypothesis, positing that metastasis depends on compatibility between cancer cells and the microenvironment of the target organ [5]. Dvorak subsequently incorporated inflammation, angiogenesis, and stromal remodeling into the conceptual framework of tumor development, expanding the view from cancer cells in isolation to the tumor microenvironment formed by cancer cells and stromal components [6]. In 2005, Kaplan et al. formally introduced the concept of the premetastatic niche (PMN) [7], indicating that the “soil” was no longer regarded as a passive and static recipient of disseminated cancer cells, but as a dynamic ecosystem that can be remotely preconditioned by the primary tumor, further selected and remodeled by disseminated cells, and coevolve with metastatic lesions. With advances in multiregional sequencing and phylogenetic analysis, metastasis is increasingly recognized as a dynamic process characterized by branching evolution and clonal selection [8, 9]. Models of metastatic dissemination have also expanded beyond individual mesenchymal‐like cells to encompass collective migration and phenotypic plasticity [10, 11].

Recent advances in multiomics profiling and preclinical modeling have further revealed the complexity of metastasis. An emerging view holds that metastatic progression is jointly shaped by genetic drivers, nongenetic plasticity, and the organ microenvironment. While metastases generally inherit the major driver alterations of their primary tumors, they may also acquire additional genomic changes under therapeutic and organ‐specific selective pressures. Moreover, no single metastasis‐driving gene appears to be universally applicable across cancer types [12, 13, 14, 15]. The major conceptual and mechanistic milestones in metastasis research are summarized in Figure 1.

**FIGURE 1:**
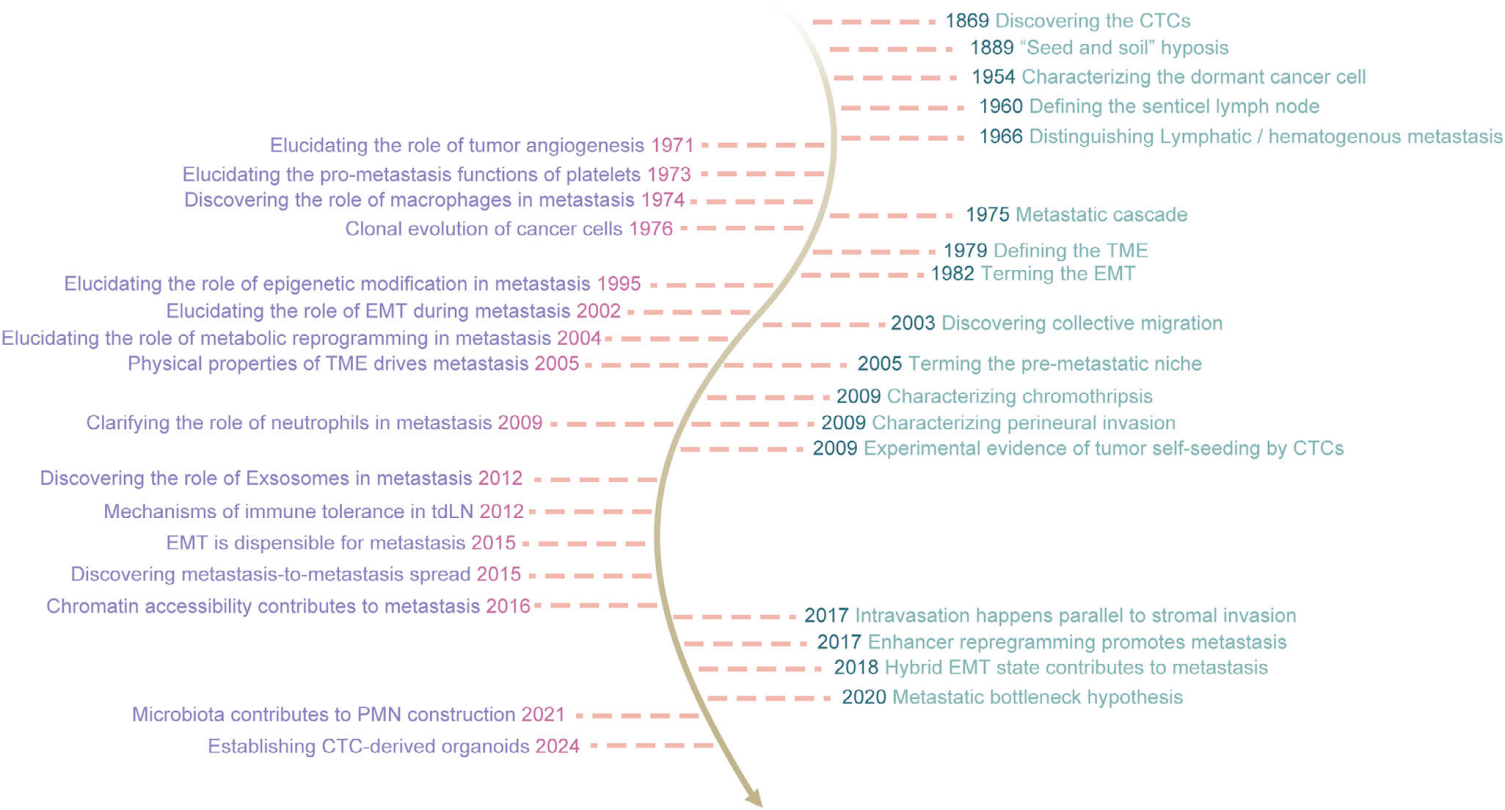
A timeline of key milestones in metastasis and mTME research. This timeline spans from 1869 to 2024, illustrating pivotal discoveries and theoretical proposals. The left side of the timeline shows the important mechanisms underlying cancer cell metastasis that have been identified, whereas the right side shows the proposal and discovery of concepts and hypotheses related to cancer metastasis. *Abbreviations*: CTC, circulating tumor cells; EMT, epithelial–mesenchymal transition; PMN, premetastatic niche; TME, tumor microenvironment.

Metastasis is now recognized as a multifactorial and inefficient process. Evidence from diverse preclinical models indicates that only a minute fraction of MTCs leaving the primary site ultimately form overt metastases. This suggests a vulnerability during dissemination, which provides a potentially exploitable window for therapeutic intervention. The vasculature, lymphatics, and nerves are the broadly shared routes of dissemination across cancer types. While these routes differ substantially, recurring principles governing the interactions between MTCs and route‐specific local microenvironments have emerged, creating opportunities to develop broadly applicable antimetastatic strategies. To characterize these interactions, we propose the concept of the migratory tumor microenvironment (mTME). The mTME includes microenvironments during MTC detachment from the primary site, dissemination through the blood, lymphatic, or nervous system, and extravasation and colonization. Recent single‐cell transcriptomic studies have begun to delineate the complex interactions between MTCs and the mTME, revealing marked spatiotemporal and intercellular differences between dissemination routes and primary or metastatic tumor microenvironments. For example, the cellular composition and transcriptional states of samples obtained during hematogenous dissemination differ substantially from those of primary and metastatic lesions [16]. During lymphatic dissemination, the immune microenvironment and cancer cell survival programs likewise undergo pronounced stage‐dependent changes [17]. These spatiotemporal distinctions, which also encompass the PMN and the established metastatic niche, are summarized in Table 1.

Against this background, this review is organized following the spatiotemporal trajectory of cancer cells from evolution within the primary tumor through dissemination to metastatic colonization. We first review differences in driver mutations, copy‐number alterations (CNAs), chromosomal instability (CIN), and chromatin accessibility during MTC metastatic evolution, illustrating how cancer‐cell heterogeneity provides a selectable repertoire of metastatic states. We discuss how interactions within the primary tumor microenvironment induce and select metastatic phenotypes. We subsequently focus on the migratory behavior and interaction patterns of cancer cells within the mTME, with particular emphasis on the shared and route‐specific survival mechanisms associated with hematogenous, lymphatic, and perineural dissemination. Using the liver, lung, and brain as representative target organs, we further examine the establishment and remodeling of organ‐specific metastatic niches. Finally, we discuss the intrinsic challenges facing metastasis research and evaluate the current advances and limitations in metastasis‐oriented diagnostic stratification and therapeutic interventions.

## 2 Genomic and Chromosomal Evolution From the Primary Tumor to Metastases

The evolution of MTCs is not a linear accumulation of genetic changes acquired during dissemination but rather a multistage process shaped by selection, adaptation, and remodeling.

### 2.1 Genomic Evolution

MTCs undergo a multistage remodeling process during the selection of subclones within the primary tumor, their propagation through dissemination bottlenecks, and ongoing evolution during metastatic spread. Research has proposed that the driver events and the chromosomal framework that determine the core malignant phenotype may be established in the primary tumor. Subclones with dissemination potential then expand locally. During migration, the exposure to distant organ microenvironments, and therapy, some are eliminated whereas others continue to evolve through point mutations, CNAs, whole‐genome duplication (WGD), structural variants (SVs), and extrachromosomal DNA (ecDNA). Therefore, while primary and metastatic lesions may share a stable ancestral genetic foundation, substantial branch‐specific differences may arise across patients, cancer types, routes of dissemination, and treatment stages [12, 18]. Data from animal models have further suggested that cancer cells may leave the primary lesion early in disease, whereas single‐cell lineage tracing findings revealed pre‐existing, heritable expression state differences in metastatic competence among clones [19, 20]. These observations indicate that occasional early shedding may directly seed distant sites. Nevertheless, while stochastic or early dissemination may occur, successful colonization is not purely random: it also depends on the cancer cell genotype and interactions with cellular environments along the migratory route. This interpretation is compatible with the selection and continued evolution of metastasis‐competent cells within the primary tumor.

#### 2.1.1 Driver‐Gene Mutations

Metastasis‐initiating cells largely retain the genetic architecture of the primary tumor at the level of somatic point mutations and driver events. Metastasis‐competent subclones may expand locally, and marked clonal selection during dissemination can elevate rare or regionally dominant subclones to the common ancestor of metastatic lesions. Primary tumor alterations such as *TP53* aberrations, *MYC* amplification, and *CDKN2A* loss may increase metastatic probability by enhancing genomic instability, cellular fitness, or treatment tolerance [12]. A pan‐cancer whole‐genome comparison found only a moderate increase in mutational burden in advanced metastases relative to primary tumors, suggesting that continued accumulation of point mutations is not the dominant genomic feature of metastatic progression [15]. Matched primary‐metastasis analysis in the TRACERx non‐small cell lung cancer (NSCLC) cohort likewise showed that, while every patient harbored metastasis‐private mutations, most driver mutations were shared between primary and metastatic lesions [21]. These findings are consistent with “bottleneck model,” in which most metastatic cells descend from a common ancestor [22]. Conversely, rare subclone sequencing studies in breast cancer reported that most putative metastasis‐driving mutations accumulated in recurrent and late metastatic lesions and were undetectable in the primary tumor [15, 23, 24]. Newly detected mutations in metastases may therefore reflect pre‐existing, low‐frequency metastasis‐competent populations missed by limited primary tumor sampling, alterations gradually acquired during dissemination, or variants selected under therapeutic pressure.

#### 2.1.2 Epigenetic Evolution

In addition to genomic alterations, epigenetic regulation also influences the evolutionary routes from primary tumor to metastasis and is a major determinant of subpopulations with distinct dissemination potential. In a murine triple‐negative breast cancer model, genome‐wide hypomethylation was found to disrupt the blood–brain barrier (BBB) through the KDM6A–matrix metalloproteinase (MMP) 3 axis and accelerated subsequent brain metastasis [25]. Some epigenetic changes may arise early during primary tumor development and establish a permissive background for later dissemination. Hypermethylation of the proximal *HNF4A* promoter was shown to suppress *HNF4A* transcription, resulting in decreased *HNF4A* expression, as shown in pancreatic intraepithelial neoplasia in KPC mice and Stage I human pancreatic ductal adenocarcinoma (PDAC), which results in increased cancer cell proliferation, clonogenicity, stemness, and invasion [26]. In an organoid model derived from primary and metastatic KPC tumors, integrated chromatin immunoprecipitation‐sequencing (ChIP‐seq), assay for transposase‐accessible chromatin using sequencing (ATAC‐seq), and RNA‐sequencing showed that metastatic regulatory loci were already accessible in normal pancreatic tissue. Metastasis‐associated transcriptional changes were driven predominantly by enhancer reprogramming that activated FOXA, GATA, and related transcription factors, induced a retrograde developmental transition toward embryonic foregut endoderm, and increased metastatic capability [27].

Together, these studies suggest that metastasis‐promoting mutations and epigenetic alterations may already be present in primary tumors. While most driver mutations and epigenetic programs seem to remain shared with the primary lesion, selection during dissemination progressively enriches malignant subpopulations with greater metastatic competence.

### 2.2 Chromosomal Evolution

#### 2.2.1 Chromosomal Structural Variation

Compared with data on point mutations, evidence for the evolution of chromosomal architecture during metastasis is more consistent. Chromosomal alterations are enriched during metastatic progression across multiple cancer types and exceed changes in point mutation burden. The MSK‐MET study found that the fraction of genome altered (FGA) was significantly higher in metastases than in primary tumors across 16 cancer types, and WGD was more frequent in metastases from seven cancer types. CIN was significantly associated with metastatic burden in prostate adenocarcinoma, lung adenocarcinoma, and hormone receptor‐positive/HER2‐positive breast ductal carcinoma [28]. Pan‐cancer genomic comparisons showed that while chromosome arm karyotypes were broadly similar between most primary and metastatic tumors, SV burden was generally higher in metastases across 15 cancer types. In prostate cancer, chromothripsis and other complex rearrangements increased by more than threefold in metastases compared with primary tumors, implicating focal amplification, deletion, and rearrangement in metastatic progression [15]. Longitudinal multisite sampling has provided more direct evidence of ongoing CNA evolution. Across serial and spatially distinct samples, the increase in FGA from primary to metastatic tumors substantially exceeded that in tumor mutational burden (20.6 vs. 6.4%), among tumors without WGD in the primary lesion, 28.5% acquired WGD in metastases. Approximately 10% of patients also acquired 9p loss during metastatic progression [29]. Collectively, these studies indicate that metastatic genome evolution is dominated by copy number and chromosome‐scale remodeling rather than continued accumulation of point mutations.

The levels of CNA and CIN are also associated with the route of dissemination. Paired sequencing in gastric cancer showed that hematogenous metastases more often displayed increased CIN and CNA burden, whereas peritoneal and ovarian metastases more frequently remained chromosomally stable and continued to evolve through newly acquired somatic driver mutations [30]. In advanced NSCLC, both the primary tumor subpopulations that disseminated beyond the thorax and their metastatic descendants carried a higher somatic CNA burden, whereas subpopulations confined to intrathoracic spread showed no comparable enrichment [31]. CIN appears to be an important component of metastatic heterogeneity. Whether it confers a realized metastatic advantage may depend on cancer type, dissemination route, and the selective pressures within the target organ.

ecDNA represents another highly dynamic form of focal amplification. Because ecDNA lacks centromeres and segregates unevenly during mitosis, it can rapidly generate intercellular differences in the copy number of oncogenes and regulatory elements. A pan‐cancer analysis of 8060 newly diagnosed primary tumors, untreated metastases, and heavily pretreated advanced tumors found significantly higher ecDNA detection rates in advanced tumors and untreated metastases than in primary tumors. In longitudinal paired analyses, 54.5% of baseline ecDNA structures had related amplicons in subsequent samples, indicating persistent selection and expansion of dominant ecDNA‐bearing clones during progression [32]. A study of 14,778 patients detected ecDNA in 17.1% of tumor samples. Furthermore, ecDNA was significantly associated with metastasis and increased with disease stage. Moreover, ecDNA‐borne immunoregulatory genes correlated with reduced intratumoral T cell abundance, suggesting a role in immune escape during metastasis [33]. By contrast, lower ecDNA positivity and fewer ecDNA events were detected in glioblastoma, sarcoma, head and neck cancer, and ovarian cancer, underscoring strong tissue and treatment context dependence [32]. In general, research on ecDNA in metastasis remains at an early stage, and further studies are required to establish its causal and regulatory roles across tissue and genomic contexts.

#### 2.2.2 Evolution of Chromatin Accessibility

The evolution of chromatin accessibility occurs in response to both the pre‐existing, selectable heterogeneity within the primary tumor and the dynamic reprogramming jointly imposed by the environment and cell state during metastasis. A single‐nucleus multiomics atlas of more than 1 million cells across 11 cancer types revealed coordinated differences in accessible regions, transcription factor motifs, and regulons between primary and metastatic samples [34]. Primary tumor atlases likewise showed that distal accessibility patterns distinguish malignant cell states and correlate with clinical outcome, indicating that some plasticity is established before dissemination [35]. Functional studies have shown that the NFIB transcription factor induces widespread chromatin opening in small cell lung cancer and activates neuronal development, motility, and adhesion programs that promote metastasis [36]. Distant PDAC metastases also undergo large‐scale epigenomic reprogramming and heterochromatin loss linked to metabolic state [37]. Thus, primary tumor heterogeneity supplies selectable initial states, whereas the metastatic microenvironment further induces or stabilizes new accessibility programs.

Changes in chromatin accessibility closely reflect the stage‐specific requirements of MTCs during metastasis. In a murine lung cancer model, acquisition of a metastatic phenotype was accompanied by progressive loss of normal differentiation features and increased accessibility at loci associated with epithelial–mesenchymal transition (EMT) and extracellular matrix (ECM) remodeling [38]. In lung adenocarcinoma, H2A.Z‐loaded nucleosomes were shown to occupy enhancers of prometastatic genes. Activation of RAS/MAPK and TGF‐β signaling evicted these nucleosomes, increased enhancer accessibility, and induced EMT transcription factors and profibrotic microenvironmental mediators [39]. Temporal ATAC‐seq in a murine osteosarcoma lung metastasis model showed that early metastatic cells preferentially open chromatin regions related to fluid shear stress (FSS), apoptosis, and escape from senescence, thereby supporting survival under microenvironmental stress. At later stages, accessible chromatin becomes enriched at genes involved in ECM remodeling, bone development, and vasculogenesis, supporting the growth of large lesions. These sequential programs coordinate stress survival, microenvironmental interactions, migration, and colonization [40].

In summary, genomic and chromatin remodeling are central molecular events in metastasis. Most metastatic lesions inherit the trunk driver events of the primary tumor. CNA, WGD, SVs, and ecDNA generate heritable structural and gene dosage heterogeneity, whereas changes in chromatin accessibility provide faster and partly reversible cell state plasticity. During progression, dissemination routes, physicochemical and metabolic conditions in target organs, immune surveillance, and therapy jointly select and reshape these variants, highlighting the central role of metastatic environments in tumor evolution. We discuss these topics in later sections.

## 3 Primary Tumor Microenvironment

The primary TME is a continuously evolving ecosystem shaped by cancer cell expansion, abnormal perfusion, tissue‐mechanical remodeling, and inflammation. Tumor cores, invasive fronts, and specialized local regions, including perivascular, lymphatic, and perineural interfaces, differ in biochemical conditions, nutrient supply, and intercellular signals and thereby induce or select cancer cell subpopulations with distinct metastatic fitness. Metabolites and secreted factors released by cancer cells reciprocally remodel stromal, immune, vascular, and neural components, establishing bidirectional coevolution [41].

### 3.1 Primary TME‐Driven Metastatic Evolution

Abnormal vascular perfusion, cellular nutrient consumption, and diffusion limits jointly establish spatial gradients within primary TME. Oxygen tension, glucose, amino acids, extracellular pH, lactate, and other biochemical variables change asynchronously across tumor cores, margins, stromal interfaces, and perivascular regions, imposing selective pressures on malignant cells and influencing their subsequent fates [42, 43]. In poorly perfused, hypoxic regions, HIF signaling is an important driver of the local invasive phenotype of MTCs. In breast cancer cells, HIF‐1α and HIF‐2α upregulate ADAM12, promotes HB–EGF shedding and EGFR–FAK activation, enhancing MTC motility and basement membrane penetration [44]. Hypoxia also promotes MTC clustering and collective intravasation, whereas improved perfusion and reduced hypoxia decrease the entry of MTC clusters into the circulation [45]. Hypoxic colorectal cancer (CRC) cells release IL‐8, which induces EMT in neighboring normoxic cells through NF‐κB/p65 and enhances their invasion and metastatic competence [46]. Hypoxia‐fate mapping further showed that cells previously exposed to hypoxia acquire a reactive oxygen species (ROS)‐tolerant state after leaving hypoxic regions, with increased invasion, intravasation, and circulatory survival [47]. Spatial transcriptomics revealed that severely hypoxic cells retain HIF, NF‐κB, and MUC1‐C‐associated transcriptional programs after reoxygenation and maintain superoxide dismutase expression to reduce oxidative injury in the circulation [48]. Hypoxic regions also often accumulate lactate. Tumor‐derived lactate released under hypoxia can be taken up by normoxic CRC stem‐like cells and enhance oxidative phosphorylation (OXPHOS) through PGC‐1α, increasing bioenergetic capacity and metastasis [49]. In human intestinal tumor organoids, lactate also increases histone acetylation and activates MYC, suppressing cancer stem cell (CSC) differentiation and driving differentiated cancer cells back toward a proliferative CSC state, thereby expanding the stem‐like fraction of MTCs [50].

Nutrient limitation within primary tumors further selects metabolically advantaged MTCs. In glucose restricted regions, Snail suppresses PFKP and redirects MTC metabolism from glycolysis to the pentose phosphate pathway, preserving NADPH production and oxidative stress tolerance [51]. Under amino acid and growth factor deprivation, mTORC1 inhibition promotes TFEB nuclear translocation, enhances endolysosomal recycling and cell surface exposure of MT1‐MMP, and increases invadopodial activity. Together with TKS5‐dependent invadopodium formation, these changes accelerate ECM degradation and invasion [52, 53]. In severely nutrient depleted regions, MTCs can convert ECM into a nutrient source. Invasive breast cancer cells use PAK1‐dependent macropinocytosis to internalize and degrade ECM and exploit tyrosine catabolism and fumarate production to sustain growth and migration [54].

In general, the primary TME may select and reinforce metastatic phenotypes of MTCs. Hypoxia, acidosis, and metabolic stress act through metabolite and cytokine exchange, connecting invasive states acquired in the primary tumor with subsequent intravasation, survival in the circulation, and distant colonization.

### 3.2 Stroma‐Derived Signals Shaping Dissemination Routes

Mechanical forces exerted by the ECM surrounding the primary tumor directly regulate cancer cell state and can precondition cells before they enter the circulation. Matrix stiffening drives EMT and invasion through the TWIST1–G3BP2 axis [55]. It can also induce stemness and chemoresistance by promoting TAZ–NANOG liquid–liquid phase separation and consequent SOX2 and OCT4 transcription, or by activating YAP and suppressing the proapoptotic factor BMF [56, 57]. ECM‐imposed mechanical stress further drives stem‐like cancer cells into quiescence. After these cells traverse a stiff, migration‐restrictive ECM and the mechanical constraint is relieved, they resume proliferation while retaining high invasiveness and resistance to anoikis [58, 59]. At the invasive front, spatial confinement activates Rho–ROCK–Myosin II signaling, induces a mesenchymal‐to‐amoeboid transition, and establishes mechanical memory through JAK–STAT signaling [4]. Consequently, even after leaving the rigid primary TME, cancer cells may retain high actomyosin contractility and apoptosis resistance, increasing survival during dissemination [60]. At convex, highly curved ECM fibers contacting the plasma membrane, ZMYM3‐dependent cortactin transcription, and caveolae‐guided cortactin accumulation activate ARP2/3‐mediated F‐actin polymerization, assembling the core scaffold of the invasive synapse [61].

Cellular components of the primary tumor stroma also actively remodel metastatic phenotypes. Fibroblasts can directly facilitate early lymphovascular invasion. Cancer‐associated fibroblasts (CAFs) in the primary TME express SOD3 to remove extracellular ROS and maintain local redox balance, while secreting VEGFA to activate VEGFR2‐dependent angiogenesis and lymphangiogenesis, thereby creating routes for distant dissemination [62, 63]. PDGFRα^+^ ITGA11^+^ CAFs use CHI3L1 to realign matrix fibers and ITGA11–SELE signaling to activate lymphatic endothelial cells (LECs), promote lymphangiogenesis, and facilitate MTC intravasation [64]. Fibroblasts can also take up LINC00665 carried by bladder cancer extracellular vesicles (EVs) and secrete HGF; HGF then increases cancer cell LINC00665 through c‐MYC, forming a self‐amplifying loop that promotes lymphangiogenesis and lymph node (LN) metastasis [65]. In PDAC, pancreatic stellate cells release alanine through autophagy, providing carbon for the tricarboxylic acid cycle and lipid synthesis and enabling metastatic subpopulations to switch fuel sources before dissemination [66].

At the tumor–nerve interface, MTCs first establish neurotropism through axon‐guidance and neurotrophic signals. PDAC cells engage PLXND1 on sensory neurons through SEMA3D, promoting neurite outgrowth and neurotropic cancer cell migration, encirclement, and penetration of the epineurium [67]. In gastric cancer, NGF–TRK‐dependent expansion of calcitonin gene‐related peptide (CGRP)^+^ sensory nerves attracts peptidergic nociceptor innervation and markedly promotes cancer cell migration toward dorsal root ganglia (DRG) [68]. Sensory neuron‐derived substance P can induce death in a small fraction of MTCs through TACR1; the released single‐stranded RNA then activates a TLR7‐dependent prometastatic program in neighboring MTCs [69]. Cancer‐associated neurons can also transfer their mitochondria to adjacent MTCs, increasing bioenergetic capacity and resistance to oxidative and mechanical stress [70]. Stromal cells subsequently stabilize this prometastatic niche. Schwann cells (SCs), the most abundant stromal cells of the peripheral nervous system, connect tumor‐driven remodeling to the neural matrix. SCs enhance PDAC cell proliferation and migration toward nerve fibers through midkine–LRP1 signaling [71]. In highly innervated lung cancers, SC‐derived CXCL5 activates CXCR2 to promote EMT and local tumor invasion and dissemination [72]. MTC‐derived NGF and galanin, together with the transcription factor EGR2, activate SC ATG7‐dependent autophagy, thereby promoting SC migration toward MTCs, axonal guidance, neurotropic MTC motility, deposition of a proregenerative ECM, and metastatic progression [73, 74]. The reciprocal neurotrophic, glial, stromal and immune interactions within the perineural microenvironment are summarized in Figure 2.

**FIGURE 2:**
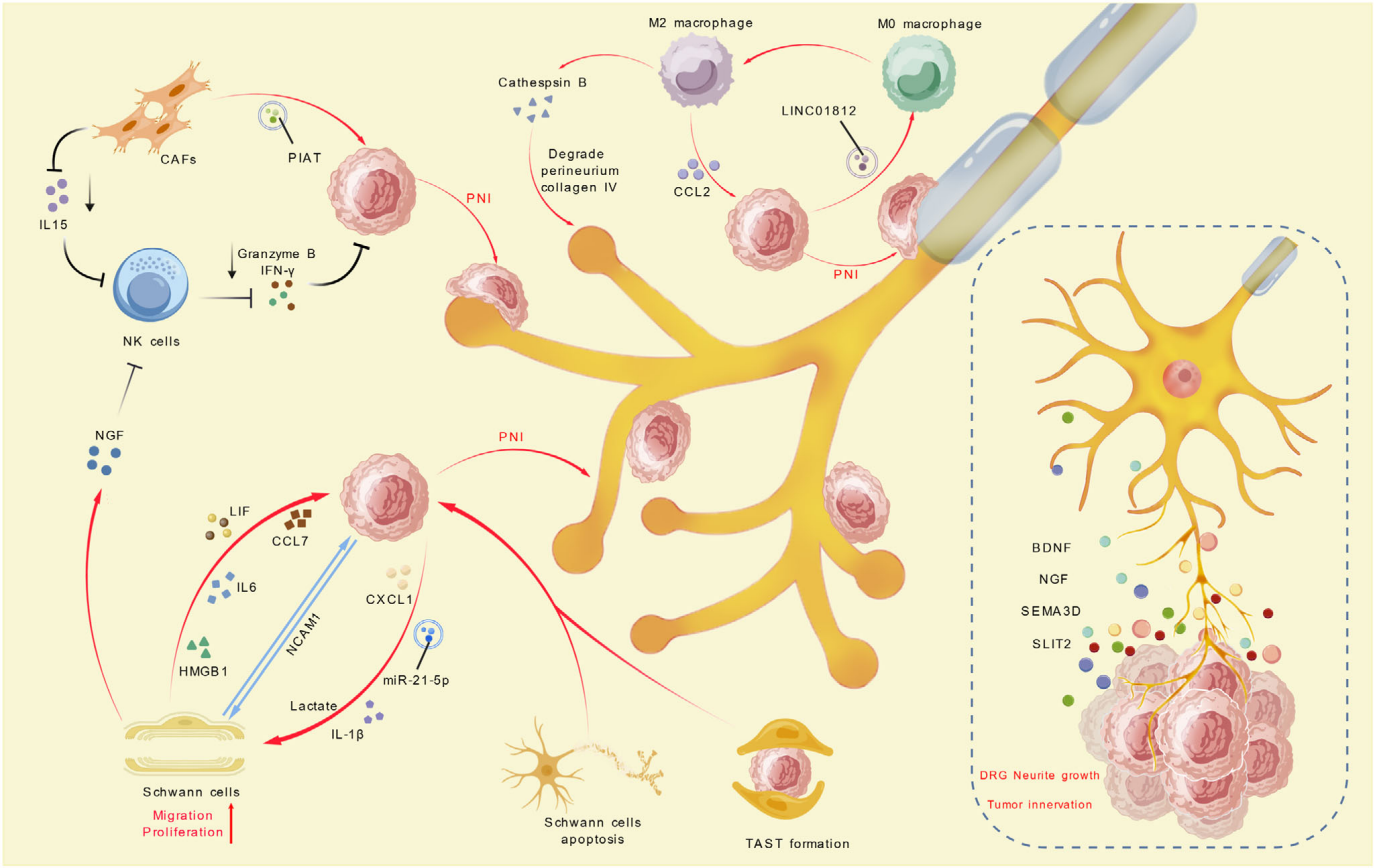
Mechanism diagram of MTC–perineural microenvironment interactions. Primary tumor cells inducing PNI by secreting neurotrophins, which stimulate DRG neurite growth and tumor hyperinnervation. Stromal cells along the PNI route, including CAFs and SCs, provide mechanical support, induce MTC metabolic reprogramming, secrete immunosuppressive molecules, chemokines, and EVs that promote PNI. Antitumor immunity is consequently impeded, evidenced by the inhibition of NK cells and the polarization of M2 macrophages. *Abbreviations*: BDNF, brain‐derived neurotropic factor; CCL2, C–C motif ligand 2; CCL7, C–C motif ligand 7; CXCL1, C–X–C motif ligand 1; LIF, leukemia inhibitory factor; NGF, nerve growth factor; PIAT, PNI‐associated transcript; PNI, perineural invasion; SEMA3D, semaphorin 3D; SLIT2, slit guidance ligand 2; TAST, tumor‐activated Schwann cell tracks. Created with BioGDP.com.

### 3.3 Immune‐Derived Signals Selecting MTC States

The immune milieu of the primary TME imposes a key selective pressure that can reshape clonal composition in both primary and metastatic lesions by eliminating susceptible tumor subpopulations. For example, samples from untreated early‐stage NSCLC showed ongoing immunoediting within immune infiltrated regions through loss of human leukocyte antigen (HLA) heterozygosity, depletion of expressed neoantigens, and promoter methylation of neoantigen‐bearing mutant genes [75]. Among long‐term survivors of pancreatic cancer, stronger T cell activity in primary tumors was associated with depletion of neoantigens and reduced genetic heterogeneity in recurrent tumors [76]. These findings suggest that MTCs successfully establishing metastases have survived immune selection in the primary TME by acquiring immunosuppressive or immune‐evasive properties.

Cancer cells accordingly evolve multiple mechanisms of immune evasion. For instance, MTC can escape macrophage phagocytosis through YAP–TEAD–CD24 signaling [77]. In the peritumoral stroma of CRC, a subset of mature regulatory dendritic cells (mregDCs) recruits and activates regulatory T cells (Tregs), which restrict mregDC migration and transport of tumor antigens to draining mesenteric LNs. This perilymphatic Treg–mregDC niche suppresses the initiation of adaptive immunity [78]. VEGF‐C‐induced lymphangiogenesis within tumors and tumor‐draining LNs (tdLNs) promotes tumor antigen uptake and cross‐presentation by LECs, induces immune tolerance, and drives CD8^+^ T cell dysfunction and deletional apoptosis [79]. Immune remodeling within primary TME therefore not only suppresses antitumor immunity but can directly confer invasive capacity, facilitate intravasation, enhance circulatory survival, and continuously regulate dissemination.

In addition, immune cells within primary TME enhance cancer cell invasion. Breast cancer cell‐derived CSF‐1 and macrophage‐derived EGF establish a positive chemotactic feedback loop that drives coordinated migration of both cell types toward blood vessels [80]. Direct macrophage–MTC contact also activates Notch1–Mena^INV^ signaling in MTCs, inducing invadopodia and transendothelial migration [81]. MTCs further amplify these invasive programs by actively reprogramming myeloid cells. Highly metastatic cells secrete mesothelin and induce macrophage production of VEGFA and S100A9, which promotes neutrophil extracellular trap (NET) formation, strengthens MTC adhesion to endothelium, facilitates intravasation, and increases distal metastasis [82]. In another PDAC model, MLKL‐driven necroptosis both induced macrophage‐dependent CD47 upregulation on MTCs and triggered formation of macrophage extracellular traps (METs). METs promoted EMT progression and ICAM‐1 expression through CXCL8 and degraded ECM, integrating antiphagocytic signaling, invasion, and endothelial adhesion into a continuous program of liver metastasis [83].

Immune cells help construct the gateways used for hematogenous and lymphatic intravasation. In breast cancer, Mena‐expressing cancer cells, Tie2^high^ VEGF^high^ macrophages, and vascular endothelial cells form tumor microenvironment of metastasis doorways that permit MTC entry into blood vessels. In PDAC, MLKL‐driven necroptosis recruits macrophages, enhances IL‐6‐associated CD47 “don't eat me” signaling, and induces MET formation; MET‐derived MMP12 activates CXCL8, whereas MMP2/9 degrades ECM, thereby facilitating transendothelial migration [83]. Through CSF‐1–CSF‐1R signaling, MTCs induce macrophage release of VEGFA, causing focal and transient opening of endothelial junctions at tumor microenvironment of metastasis doorways and creating a spatiotemporal window for intravasation. Blocking this pathway reduces MTC generation and distant dissemination [84, 85]. Bladder cancer cells recruit CXCR2^+^ neutrophils through CXCL1/CXCL8; these cells secrete VEGFA and MMP9, promoting lymphangiogenesis, ECM degradation, and LN metastasis [86]. Tregs activate noncanonical NF‐κB signaling in MTCs and LECs, enhancing translymphatic endothelial migration and inducing LEC expression of SOX18, FLRT2, and myeloid chemokines. It also converts VE‐cadherin organized, zipper‐like LEC junctions into discontinuous button‐like junctions, facilitating effector T cell egress from tumor and depleting local antitumor immunity [87].

### 3.4 Coevolution of the Microbiota and MTCs

Through community composition, intratumoral colonization, and interorgan dissemination, the microbiota can connect the acquisition of metastatic phenotypes in the primary tumor with circulatory survival and distant colonization. Dysbiosis induces inflammation and stromal remodeling in the primary organ; intratumoral microorganisms directly reprogram cancer or immune cells; and some bacteria and fungi codisseminate with MTCs and enhance their survival in the circulation.

Dysbiosis can promote a metastatic phenotype before tumor formation or dissemination. In breast cancer models, gut dysbiosis induces mammary inflammation, myeloid‐cell infiltration, and collagen deposition and increases MTC numbers and early dissemination independently of primary tumor size [88]. After enterotoxigenic *Bacteroides fragilis* (ETBF) colonizes the intestine or mammary ducts, *B. fragilis* toxin (BFT) activates β‐catenin and Notch1, allowing MTCs to retain migratory, invasive, and tumorigenic advantages after only transient ETBF exposure [89]. Thus, remote microbiome alterations may select metastatic subpopulations indirectly through inflammation and stromal remodeling or imprint durable nongenetic phenotypic memory.

Within primary tumors, intratumoral microorganisms can directly induce epithelial–mesenchymal plasticity, stemness, and cytoskeletal remodeling. *Fusobacterium nucleatum* adheres to and invades CRC cells, inducing IL‐8 and CXCL1 release and directional MTC migration [90]. It also activates YAP and suppresses the FOXD3–METTL3 axis, sustaining cytoskeletal remodeling and promoting liver and bone metastasis [91]. DNA damage and the senescence‐associated secretory phenotype induced by colibactin‐producing *Escherichia coli* (CoPEC) drive EMT in both infected and uninfected cells, increase CD133, OCT3/4, and NANOG expression, and generate drug‐resistant CSC subpopulations [92]. BFT‐1 secreted by ETBF also binds and stabilizes NOD1 and activates NOTCH1–HEY1 signaling through GAK‐mediated NUMB degradation, expanding ALDH^+^ breast CSCs [93]. Focal microbial colonization can therefore amplify a restricted bacterial niche into widespread epithelial–mesenchymal plasticity and stemness remodeling across the primary tumor.

Intratumoral microorganisms also regulate the primary niche through metabolic and immune remodeling. CoPEC creates focal microdomains enriched in glycerophospholipids and lipid droplets; these regions show reduced CD8^+^ T cell infiltration and IFN‐γ production, indicating that bacteria can simultaneously provide metabolic buffering to MTCs and promote immune exclusion [94]. In breast cancer, *F. nucleatum* selectively colonizes tumors when Fap2 recognizes Gal–GalNAc on MTCs and reduces tumor‐infiltrating T cells [95]. In gastric cancer, the same organism recruits protumor neutrophils and establishes an immunosuppressive myeloid niche [96]. Intracellular *F. nucleatum* in esophageal squamous cell carcinoma upregulates cancer cell PD‐L1 while suppressing T cell proliferation and cytokine secretion, thereby coupling immune escape, treatment resistance, and metastatic selection [97].

Microbial effects can extend to later stages of the metastatic cascade. Intracellular bacteria in breast cancer can be carried into the circulation by MTCs and remodel the cancer cell actin cytoskeleton to increase resistance to FSS, thereby promoting lung dissemination and colonization [98]. In head and neck squamous cell carcinoma (HNSCC), *F. nucleatum* activates the TLR4–MYB–ESPN axis to increase filopodia, endothelial adhesion, and transendothelial migration, directly linking cytoskeletal remodeling in the primary tumor to subsequent extravasation [99]. *E. coli* within primary CRC can first disrupt the gut vascular barrier and reach the liver, where it induces a PMN before facilitating MTC colonization [100]. In lung adenocarcinoma, lactate produced by intratumoral *Staphylococcus nepalensis* and *Staphylococcus capitis* induces an MCT1 and NDRG1‐associated pseudohypoxic program in cancer cells and enhances metastatic competence [101]. Thus, microbiota are not isolated carcinogenic factors at the primary site; through inflammatory, metabolic, cytoskeletal, and immune signals, they connect primary tumor adaptation with survival in the circulation and distant colonization.

In summary, spatial gradients and multicellular interactions within the primary TME jointly drive metastatic evolution. Hypoxia, acidosis, and nutrient deprivation first induce or select subpopulations with epithelial–mesenchymal plasticity, metabolic fitness, and resistance to oxidative stress. Signals derived from the ECM, CAFs, and neural glia then stabilize invasive states and direct cells toward blood vessels, lymphatics, or nerves. Remodeling by macrophages, neutrophils, and Tregs further amplifies dissemination. Microbiota‐derived signals further sustain and amplify metastatic phenotypes from the primary tumor through circulation and colonization.

## 4 The mTME

The mTME is a transient, process‐dependent ecological interface formed by the interactions of MTCs with local physical conditions, fluid and matrix components, route‐resident cells, and recruited circulating cells along a defined dissemination pathway. The mTME encompasses the environments encountered from MTC entry into a blood or lymphatic vessel, or establishment of sustained contact with a neural migratory interface, through departure from the corresponding transport structure and entry into the parenchyma of a secondary tissue [102]. This definition distinguishes the mTME from other metastasis‐related environments. The primary TME principally enables acquisition of metastatic competence and prepares cells for dissemination; the mTME selects and supports cancer cells while they traverse a dissemination route; whereas the metastatic niche is defined by the sustained interactions between MTCs that have left the route and parenchymal cells in a secondary organ. All three mTME require adaptation to cell death, mechanical constraints, metabolic stress, and immune surveillance, but the mediators and molecular mechanisms that satisfy these requirements are strongly route‐specific.

It is worth noting that hematogenous, lymphatic, and perineural dissemination are not parallel routes chosen with equal probability. Route selection depends on the cancer type, the anatomy of the primary site, MTC‐intrinsic state, and remodeling of the primary TME. Table 2 summarizes the conserved and route‐specific mechanisms of cancer cells’ adaptation during migration within different microenvironments.

**TABLE 1:** Spatiotemporal distinctions of metastasis‐associated microenvironments.

TME type	Spatial pattern	Temporal pattern	Core driving mechanisms	Impact on cancer cells	References
Primary tumor microenvironment	Primary tumor site	Before dissemination of metastatic cancer cells	Bidirectional interactions among cancer cells and CAFs, TAMs, MDSCs, Tregs, endothelium, neurons, and ECM. Intratumoral hypoxia; angiogenesis, ECM degradation, and remodeling, inflammation	Inducing EMT, stemness, and invasive phenotype; disruption of the basement membrane, and promoting cancer cell detachment and invasion	[3, 103, 104]
Migratory tumor microenvironment	Blood circulation, lymphatic vessels and lymph nodes, nerve fibers, and perineural spaces	From detachment to adhesion of metastatic cancer cells	Mechanical adaptation, resistance to oxidative stress, metabolic reprogramming, immune surveillance evasion, and heterogeneous cell interactions	Screening cancer cells with high plasticity and stress resistance; enhancing the survival of cancer cells along the migration pathway	[3, 105]
Premetastatic niche	Distant target organ	Before metastatic cancer cells arrive at the metastatic site	Primary tumor‐derived EVs, soluble factors, inflammatory mediators, and metabolites act remotely on target organs	Enhancing cancer cell adhesion, extravasation, early survival, and immune evasion; determining organotropic metastasis	[106, 107, 108]
Metastatic niche	Distant target organ	During and after the arriving and colonization of metastatic cancer cells	Bidirectional remodeling of disseminated tumor cells and organ‐resident endothelial cells, fibroblasts, immune cells, glial cells	Determining whether cancer cells survive or enter dormancy or form proliferative metastases after extravasation	[109, 110, 111]

*Abbreviations*: CAF, cancer‐associated fibroblast; ECM, extracellular matrix; EMT, epithelial–mesenchymal transition; EV, extracellular vesicle; MDSC, myeloid‐derived suppressor cell; TAM, tumor‐associated macrophage; TME, tumor microenvironment; Treg, regulatory T cell.

**TABLE 2:** Conserved prometastatic programs and route‐specific mechanisms of the migratory tumor microenvironment.

	Mechanical adaptation and migration interface remodeling	Metabolic plasticity	Immune evasion and suppression	Migratory phenotype acquired by MTCs	References
Conserved prometastatic mechanisms	Cancer cells sense and adapt to the mechanical or structural constraints of their migratory pathways, converting them into signals for survival, migration, and adhesion.	Under conditions of nutritional variation, oxidative stress, and lipid metabolic pressure, cancer cells maintain energy supply and redox homeostasis.	Suppression of NK/T cell functions, recruiting or reprogramming myeloid cells and Tregs, utilizing immune checkpoints or inhibitory cytokines to establish a local immunosuppressive niche.	Higher survival ability, high plasticity, high colonization ability, and low immune visibility.	[3, 103, 112]
Hematogenous prometastatic mechanisms	FSS induction promotes antiapoptotic, glycolytic, endothelial adhesion, and invasive signaling pathways; the formation of homotypic or heterotypic CTC clusters provides mechanical adaptation.	Folate/one‐carbon metabolism, GSH production, and lactate uptake/utilization maintain NADPH and ROS clearance; iron homeostasis and lipid peroxidation tolerance resist ferroptosis.	Inhibition of NK and T cells functions through expression of immune checkpoints; platelets, neutrophils, NETs, Tregs, and MDSCs form heterotypic clusters that impair NK and CTL cytotoxicity.	Resistance to shear stress, antianoikis, antioxidation, clustering; and vascular retention.	[113, 114, 115, 116, 117, 118]
Lymphatic prometastatic mechanisms	LEC remodeling promotes cancer cell–LEC adhesion and retention in lymphatic vessels/lymph nodes; solid stress in lymph node metastases cause immune cell entry impairment through HEV remodeling.	Utilizing lymphatic GSH and fatty acids to resist ferroptosis; enhancing fatty acid uptake and FAO to promote lymph node colonization.	LECs, Tregs, CAFs, and suppressive monocytes constitute the immunosuppressive niche of metastatic lymph nodes, inducing T cell exhaustion and regional immune tolerance.	Ferroptosis resistance, regional immune tolerance, lymph node colonization, and the ability to continue dissemination via lymph nodes.	[119, 120, 121, 122, 123, 124, 125]
Perineural prometastatic mechanisms	Schwann cells provide a physical scaffold and mechanical guidance for the directional migration of cancer cells along nerves; TAM degrade collagen, weakening the perineurial barrier.	Schwann cell, monocyte, and macrophage form a positive feedback loop that induces local immunosuppression.	CAF‐derived paracrine effects impair NK cell cytotoxicity; Schwann cell–TAM interactions induce local immunosuppression.	PNI, matrix barrier penetration, and local immune evasion.	[70, 126, 127, 128, 129, 130, 131]

Abbreviations: CAF, cancer‐associated fibroblast; CTC, circulating tumor cell; CTL, cytotoxic T lymphocyte; FAO, fatty acid oxidation; FSS, fluid shear stress; GSH, glutathione; HEV, high endothelial venule; LEC, lymphatic endothelial cell; MDSC, myeloid‐derived suppressor cell; mTME, migratory tumor microenvironment; NADPH, nicotinamide adenine dinucleotide phosphate; NET, neutrophil extracellular trap; NK cell, natural killer cell; PNI, perineural invasion; ROS, reactive oxygen species; TAM, tumor–associated macrophage; Treg, regulatory T cell.

### 4.1 Anchorage‐Independent Survival and Anoikis Resistance

When MTCs detach from the ECM and disseminate through the circulation or body cavity, anoikis is the first major survival barrier limiting metastatic efficiency [132]. Loss of integrin‐mediated adhesion not only attenuates survival signaling but also triggers cytoskeletal reorganization, ROS accumulation, mitochondrial and endoplasmic reticulum stress, increased replication stress, and mitotic errors. Anchorage‐independent survival therefore represents an integrated adaptation to detachment‐induced mechanical changes, multiple cellular stresses, and genomic instability. Current evidence indicates that MTCs acquire anoikis resistance through multiple mechanisms that reprogram adhesion signaling, preserve organelle homeostasis, and protect genome integrity, thereby facilitating metastasis.

Adaptive remodeling of adhesion signaling is exemplified by the adhesion‐to‐suspension transition (AST). MTCs upregulate hematopoietic transcriptional regulators, including IKZF1, NFE2, BTG2, and IRF8, while suppressing YAP–TEAD and integrin/ECM programs, thereby enabling spontaneous matrix detachment. Although matrix adhesion is reduced, intercellular adhesion is retained during AST, allowing suspended cells to form homotypic aggregates and collectively resist anoikis [112]. Additionally, microenvironment‐derived MFG‐E8 binds integrins on MTCs, reactivates and stabilizes their active conformation, and mimics adhesion‐dependent survival signaling to reinforce anoikis resistance [133].

In parallel with adhesion signaling adaptation, maintenance of organelle homeostasis constitutes another core layer of anoikis resistance. Cytoprotective autophagy buffers suspension‐induced stress by sustaining energy supply and organelle quality control. In prostate cancer cells in three‐dimensional suspension culture, the ATF4–CEMIP–PKCα pathway was shown to relieve BCL‐2‐mediated inhibition of Beclin‐1, initiating protective autophagy and reducing anoikis [134]. Similarly, the enhanced interaction between dynamin‐related protein 1 and the endoplasmic–reticulum chaperone BiP was found to promote formation of mitochondria‐associated endoplasmic reticulum membranes. By coordinating mitochondrial fission and mitophagy, this process supports mitochondrial biogenesis and clearance of damaged mitochondria, enabling MTCs to survive detachment [135].

Preservation of genomic integrity is likewise essential for the survival of suspended MTCs, and this has been validated in mouse models. Under hypoxia, ovarian cancer spheroids in ascites exploit a noncanonical function of SAT1 to maintain H3K27ac at mitotic‐regulatory loci, thereby preventing chromosome segregation errors and mitotic catastrophe [136]. Prostate cancer cells depend on the YEATS2–NR2C2 axis to increase RAD50 transcription and DNA repair mediated by the MRN complex, preserving the survival and metastatic potential of suspended cells [137]. Therefore, genome maintenance is an important but potentially underappreciated mechanism of anoikis resistance and a candidate vulnerability of cancer cells during dissemination.

In summary, cancer cells overcome the multiple stresses caused by matrix detachment through the coordinated reprogramming of adhesion signals, adaptive protection of organelles, and maintenance of genome stability, thereby traversing the rate‐limiting step of the metastatic cascade. These anoikis‐resistance mechanisms principally apply to hematogenous, lymphatic, and transcoelomic dissemination. MTCs undergoing perineural invasion (PNI) generally remain attached to neural ECM, basement membrane, or SCs, and thus anoikis is not a major barrier in every migratory niche.

### 4.2 Mechanical Adaptation and Physical Structure Interactions Within the mTME

#### 4.2.1 Mechanical Adaptation Within the Hematogenous mTME

After entering the bloodstream, MTCs encounter membrane damage, cytoskeletal deformation, oxidative stress, and mitochondrial dysfunction induced by FSS. As FSS intensity and exposure duration increase, most mechanically fragile cancer cells undergo apoptosis. FSS can also enhance NKG2D‐dependent mechanosensing by natural killer (NK) cells and thereby promote MTC clearance [138]. A minority of mechanically resilient cells survive and acquire greater stemness, drug resistance, and metastasis‐initiating capacity, becoming tolerant of circulatory mechanical stress [139]. At the level of direct physical protection, transient high‐magnitude FSS rapidly activates RhoA and subsequent formin‐mediated F‐actin remodeling, promoting Myosin II contraction, increasing cell stiffness, and reducing plasma membrane rupture, producing rapid mechanical adaptation [114]. Microfluidic models further show that FSS upregulates TLR4 and TPPP3 and suppresses p53‐Bax‐mediated apoptosis [140]. Metabolically, physiological laminar FSS enhances glycolysis through AKT–ATOH8–HK2 signaling, whereas transient high‐magnitude shear was shown to activate folate and one‐carbon metabolism to preserve energy supply and NADPH production. Despite differences in flow pattern, both responses increased ATP availability, limited ROS accumulation, and suppressed mitochondria‐dependent apoptosis [113, 141]. Therefore, mechanotransduction‐induced metabolic and biophysical remodeling protects MTCs from immediate injury while generating prometastatic traits that facilitate subsequent extravasation and colonization.

Repeated or sustained FSS exposure enriches highly stem‐like subpopulations through selection and induction. Sustained FSS activates CXCR4–PI3K/AKT signaling and selects CD133^high^, CD44^high^, and BMI1^high^ MTCs [142]. FSS also directly induces stem‐like states: CD44 expression in HeLa cells was shown to increase with shear stress magnitude and was accompanied by chemoresistance [143]. Moreover, FSS induced the release of miR‐21‐enriched exosomes that expand the CD44^+^CD24^−^ stem‐like fraction in recipient MTCs [144]. Thus, FSS both selects pre‐existing, highly adaptable MTCs and induces stemness and biophysical plasticity through mechanotransduction. However, direct evidence that vascular wall stiffness modulates stemness is still lacking.

Nonmalignant cells within the hematogenous mTME further enhance mechanical adaptation. Heterotypic MTC clusters provide mechanical buffering and promote endothelial adhesion [138]. As MTCs roll along vessels, they generate local high‐shear vortices near the vessel wall that activate adhesion molecules and recruit platelets. Platelet coating and fibrin scaffolds attenuate shear injury and increase microvascular arrest; in vitro studies showed that platelet‐delivered tissue‐type plasminogen activator dissociated MTC clusters, suggesting that destabilizing clusters may be a feasible antimetastatic strategy [145, 146]. Clinical liquid‐biopsy studies also showed that MTCs from patients with prostate cancer frequently form heterotypic clusters with cells expressing cancer‐associated macrophage markers. These clusters display low stiffness, high deformability, and strong adhesiveness and efficiently resist shear‐induced injury [147]. Thus, cell aggregation and coating within the hematogenous mTME amplify the intrinsic gains in mechanical strength and stemness of MTCs.

#### 4.2.2 Mechanical Adaptation Within the Lymphatic mTME

Compared with hematogenous dissemination, there is limited evidence that the low shear forces within lymphatic vessels select cancer cells or directly enhance metastatic competence. Mechanical regulation and structural adaptation in the lymphatic mTME instead depend primarily on interactions between cancer cells and LECs, which promote lymphatic entry, arrest, and colonization. Single‐cell and spatial transcriptomic analyses of sentinel LNs from breast cancer showed expansion of adhesive CD200^+^ HEY1^+^ capillary‐like LECs in metastatic nodes. In response to TGF‐β and VEGF, these cells upregulated matrix Gla protein and strengthened MTC adhesion to the lymphatic wall [119]. LEC‐derived TGF‐α establishes chemotactic guidance through cancer cell EGFR–STAT3 signaling and promotes lymphatic entry [148]. Tumor‐derived EVs also induced LEC release of CXCL8 and CXCL2, promoting NET formation and deposition, which provides an adhesive scaffold for cancer cell retention and nodal dissemination [149].

Matrix stiffness at the primary site can remotely influence lymphatic dissemination. A rigid primary tumor matrix was shown to activate PIEZO1–NF‐κB signaling, increases cancer cell IL‐8 secretion, induces NET formation, and promote LEC migration and lumen formation, thereby facilitating MTC entry into the lymphatic mTME [150]. After LN metastasis formation, the solid stress generated by tumor growth compresses high endothelial venules and reduces perfusion, suppressing immune cell homing molecules such as peripheral node addressin and ICAM1. The resulting immune‐excluded niche also promotes secondary dissemination from LNs to distant organs [125]. Lymphatic dissemination therefore depends less on resistance to high shear stress than on active remodeling of LEC adhesion, LN sinus architecture, and immune cell trafficking.

#### 4.2.3 Mechanical Adaptation Within the Perineural mTME

Because the perineural microenvironment lacks a conventional lumen, cancer cells undergoing perineural migration do not experience FSS of the magnitude encountered in blood vessels. Physical contacts among the perineural matrix, nerve fibers, and cancer cells instead define the mechanical microenvironment of PNI. In breast and pancreatic cancer, PNI regions exhibit increased matrix stiffness, which activates ITGB1–FAK–YAP and PIEZO1–YAP1 signaling, promotes EMT and neurotrophic factor secretion, and enhances cancer cell adhesion to nerves, invasion, and migration along DRG neurites [151, 152]. Spatial confinement further promotes PNI. In mouse models, narrow perineural tracks were shown to activate Rho/ROCK and drive PDAC cells toward a low stiffness, highly deformable amoeboid phenotype, enabling passage through constrictions and adhesion to neurites and thereby increasing PNI efficiency [130].

Physical interactions between SCs and cancer cells likewise promote PNI. After sensing perineural matrix mechanics, SCs transmit proinvasive signals to cancer cells through c‐Jun phosphorylation [153]. Live‐cell imaging also showed that SCs repeatedly contact tumor clusters and intercalate between cancer cells, inducing membrane protrusions and guiding migration along regenerating neurites. GFAP^+^ SCs also contact MTC clusters through NCAM1, intercalate into and disperse these aggregates, and promote single cell invasion [154, 155]. In PDAC, c‐Jun‐dependent reprogramming organizes SCs into dynamic tumor‐activated SC tracks, which provide both a structural path and mechanical traction for MTC migration [131].

In summary, the physical structures and mechanical forces within mTMEs of the three dissemination routes both select cancer cells and induce prometastatic phenotypes. Hematogenous dissemination relies on adaptation to FSS, increased stemness, and protection by cell clusters to support circulatory survival. Lymphatic dissemination primarily depends on tumor–LEC interactions and solid stress‐driven remodeling that facilitates lymphatic entry and LN colonization. PNI requires cancer cells to switch migratory modes in response to matrix stiffening, spatial confinement, and SC‐mediated physical guidance.

### 4.3 Metabolic Reprogramming Within the mTME

MTCs entering different dissemination routes encounter markedly different redox and nutrient conditions. Blood imposes high oxidative stress and ROS accumulation; lymph contains abundant glutathione (GSH) and oleate and is less oxidizing; and perineural spaces support metabolite exchange among neurons, SCs, and CAFs. To survive and metastasize in these environments, cancer cells must reprogram metabolism to sustain ATP production, coordinate ROS detoxification, and preserve membrane lipid stability.

#### 4.3.1 Metabolic Reprogramming Within the Hematogenous mTME

MTCs entering the bloodstream first increase reducing capacity to remove accumulated ROS and limit oxidative injury. They enhance folate metabolism to generate NADPH or increase cytosolic and mitochondrial GSH synthesis and accumulation [115, 117, 156]. Cells undergoing AST also activate a hematopoietic‐like transcriptional program and upregulate globin genes, including HBA1 and HBA2, to reduce detachment‐induced ROS [112]. In metastatic melanoma and osteosarcoma, circulating lactate is imported through MCT1 and enters the tricarboxylic acid cycle, sustaining NADPH production and redox homeostasis [157, 158]. Clinically, elevated serum lactate and lactate dehydrogenase in patients with metastatic CRC correlate with metastatic disease and poor prognosis, supporting a role for dysregulated lactate metabolism in progression [159]. Hematogenous metabolic adaptation therefore depends on balancing reducing equivalents, mitochondrial function, and rapid energy production rather than on a single substrate.

Lipid‐metabolic remodeling further buffers oxidative stress by limiting lipid peroxidation and ferroptosis. Lactate relieves ASIC3‐mediated inhibition of ACC1/SCD1, promotes monounsaturated fatty acid accumulation, reduces lipid peroxidation, and increases ferroptosis resistance. When cancer cells leave a monounsaturated fatty acid‐rich environment, their dependence on the GPX4 antioxidant system increases [160, 161]. The lipogenic regulator SREBF2 induces transferrin expression, reduces labile iron and lipid peroxidation, and directly enhances ferroptosis resistance [162]. Lipid remodeling is not uniformly protective, however. In cancer cells derived from ovarian cancer metastases, ACSL4 promotes polyunsaturated fatty acid synthesis and incorporation into membrane phospholipids, increasing membrane fluidity and invasiveness but also heightening ferroptosis sensitivity [163]. Thus, lipid metabolism during metastasis dynamically balances energy supply, membrane plasticity, and protection from lipid‐peroxidation stress.

#### 4.3.2 Metabolic Reprogramming Within the Lymphatic mTME

Compared with blood, lymph is characterized by lower oxidative stress and labile iron and enrichment in GSH and oleate, providing MTCs with a relatively protective dissemination route [123]. Pan‐cancer multiomics analysis indicated that MTCs reduce endogenous oleate synthesis, enriching their membranes in peroxidation‐prone polyunsaturated fatty acids and increasing ferroptosis sensitivity. The oleate abundant in lymph suppresses lipid peroxidation and enhances migration and invasion, suggesting that an intrinsic lipid vulnerability may paradoxically favor lymphatic dissemination when matched to the lymphatic milieu [164]. Melanoma cells entering lymphatics exploit oleate and resist ferroptosis through ACSL3‐mediated membrane‐phospholipid remodeling [123]. During lymphatic transport and early LN colonization, cancer cells can switch from GPX4‐dependent to FSP1‐mediated ferroptosis protection and upregulate genes involved in fatty acid uptake, transport, and oxidation. This transition from glycolysis to fatty‐acid oxidation (FAO) adapts cells to the nutrient environment of LNs and supports metastatic growth [121, 165].

After LN colonization, MTCs progressively acquire cell‐intrinsic metabolic adaptations. Early LN‐resident MTCs increase CD36‐mediated lipid uptake, ATGL‐dependent lipolysis, and FAO and use FAO‐driven OXPHOS to sustain stemness and energy supply. ROS generated by OXPHOS activates the NRF2–SLC7A11–GSH axis, buffering oxidative stress and suppressing lipid peroxidation and ferroptosis [166]. By contrast, melanoma cells in established LN lesions show reduced NRF2, GCLC, and GSH and shift further toward FSP1‐dependent protection from lipid peroxidation under nodal hypoxia, thereby supporting intranodal growth [165]. These dynamic changes in GSH‐dependent redox defense likely reflect differences in tumor type, metastatic stage, and selective pressure. Metabolic therapy for lymphatic metastasis may therefore be stratified by disease stage.

#### 4.3.3 Metabolic Reprogramming Within the Perineural mTME

The perineural microenvironment remodels tumor metabolism through secretory networks, metabolite exchange, and organelle transfer. Direct neuron–tumor interactions can switch cancer cell metabolic programs and induce metastasis‐adaptive phenotypes. HNSCC cells induce neuronal CAV2 expression, and CAV2^+^ neurons redirect tumor metabolism from glycolysis toward OXPHOS. This response increases mitochondrial abundance, mitochondrial DNA content, membrane potential, and respiratory capacity and is accompanied by high tumorsphere formation and cisplatin resistance, indicating that neuron‐induced mitochondrial metabolism supports stemness and drug resistance [167]. Neurons can even transfer intact mitochondria to cancer cells through tunneling‐nanotube‐like structures. Recipient cells acquire higher OXPHOS, ATP production, GSH homeostasis, antioxidant capacity, and shear resistance and are significantly enriched in metastatic ability, demonstrating strong selection by the neural microenvironment [70].

Metabolite exchange and secretory networks within the perineural mTME likewise sustain neuroinvasive phenotypes. PNI‐associated CAFs increase glycolysis and secrete lactate; after uptake by cancer cells, lactate induces H3K18 lactylation and upregulates neural‐invasion genes such as L1CAM and SLIT1 [126]. Lactate also promotes SC dedifferentiation and HMGB1 expression, amplifying a nerve repair‐like response and strengthening SC–tumor interactions [168]. Palmitate induces an EGR2–galanin program that activates a neural‐like transcriptional state in SCs and promotes secretion of a proregenerative ECM, thereby initiating metastasis [74]. Neural glutamate selectively enhances PDAC‐cell invasion along DRG neurites through GRIN2D, whereas tumor‐derived TGFA activates EGFR on sensory nerves and promotes axonal growth, reciprocally accelerating PNI [169].

In summary, cancer cells deploy highly plastic metabolic adaptations in different dissemination routes. Hematogenous metastasis prioritizes survival under high oxidative stress, with lipid remodeling balancing ferroptosis sensitivity and membrane fluidity. Lymphatic metastasis exploits the low oxidative stress and abundant oleate of lymph, combining ferroptosis escape with an FAO‐driven metabolic switch to establish nodal lesions. PNI depends on neuron–tumor metabolic coupling and mitochondrial transfer, and local lactate and lipid signals are used to sustain invasion. These programs not only maintain energy supply and redox homeostasis but also determine MTC fate within each route‐specific microenvironment.

### 4.4 Immune Regulation Within the mTME

#### 4.4.1 Immune Regulation Within the Hematogenous mTME

During hematogenous dissemination, MTCs dynamically interact with platelets, neutrophils, lymphocytes, and coagulation factors. These interactions establish a biologically distinct hematogenous mTME that continuously remodels pericellular immunity during free circulation, intravascular migration, and the formation of circulating tumor microemboli (CTMs). The major survival mechanisms and cellular interactions that define the hematogenous mTME are shown in Figure 3.

**FIGURE 3:**
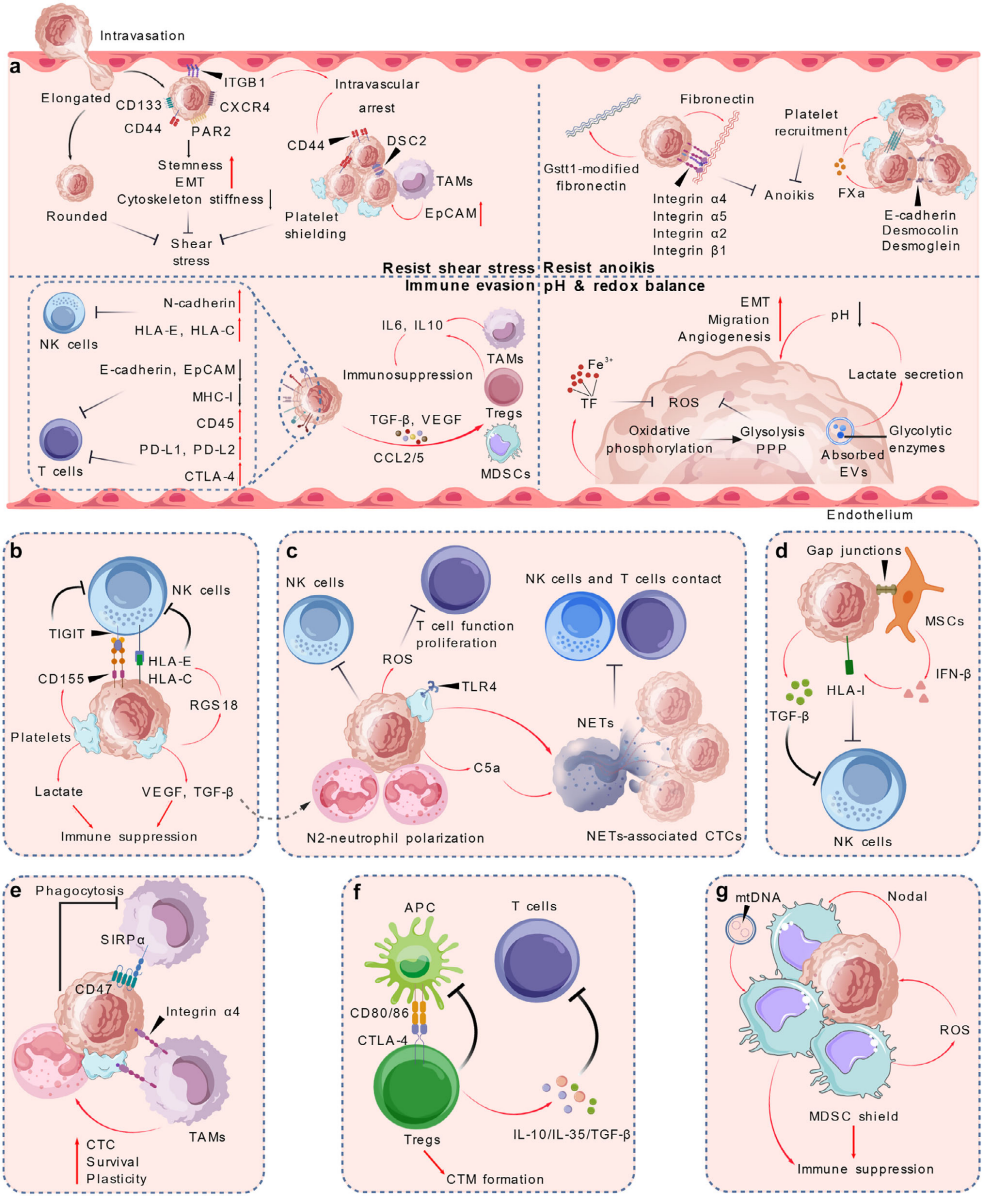
Mechanisms of MTCs survival and the cellular interactions within the hematogenous mTME. (a) Schematic representation of survival mechanisms employed by MTCs in circulation. (b–g) Key interactions between MTCs, blood, and immune cells that constitute hematogenous mTME. (b) Platelets shield MTCs to provide mechanical strength and upregulate immune checkpoints on MTCs to inhibit NK cells activity. (c) Crosstalk between neutrophils and MTCs, including the formation of NETs, shielding of MTCs, thereby inhibiting NK and T cells functions. (d) MTCs direct inhibition of NK cell function through HLA‐I molecules. (e) MTCs contact with TAMs enhance MTCs survival by inhibiting phagocytosis. (f) Tregs inhibit dendritic cells and T cells functions and participate in the formation of CTMs. (g) MDSCs shield MTCs and engage in signaling that contributes to immune suppression. Abbreviations: CTM, circulating tumor microemboli; MDSC, myeloid‐derived suppressor cell; MSC, mesenchymal stem cell; NET, neutrophil extracellular trap; NK cell, natural killer cell; ROS, reactive oxygen species; TAM, tumor‐associated macrophage; TGF‐β, transforming growth factor‐β; VEGF, vascular endothelial growth factor. Created with BioGDP.com.

During the first 24 h after MTCs enter the circulation, NK cells are the principal effectors that eliminate MTCs and therefore impose the most immediate innate immune selection [170]. MTCs evade this pressure by upregulating HLA Class I molecules as inhibitory NK cell checkpoints. Paired single‐cell transcriptomic analysis in PDAC showed that MTCs suppress distinct NK cell subsets through HLA‐E–CD94/NKG2A and HLA‐C–KIR2DL1/3, reducing granzyme release and cytotoxicity [16, 171]. Additionally, mesenchymal stromal cells in the primary tumor signal through intercellular cGAS–cGAMP–STING and IFN‐β to induce high HLA‐I expression in MTCs before intravasation, prearming them against NK cells [172]. EMT plasticity and clustering capacity further shape immune fate in the circulation. Lung cancer cells in a hybrid EMT state downregulate NK cell chemoattractants, including CXCL1/CXCL8, and upregulate B7‐H3, thereby reducing NK cell recruitment and killing [173]. MTCs that retain epithelial adhesion more readily form clusters and gain a selective survival advantage [174]. MTCs also recruit Tregs through the p38–MAX–CCL5 axis. Treg‐derived TGF‐β directly suppresses mTOR‐dependent metabolic activation and cytotoxicity in NK cells and induces further CCL5 upregulation in MTCs, establishing a positive‐feedback loop that consolidates local immunosuppression [175, 176]. Finally, MTCs can acquire CD45 from leukocyte‐derived EVs. Homotypic CD45 interactions disrupt T cell immunological synapse formation and attenuate downstream T cell receptor signaling, proliferation, and cytotoxicity [177].

After MTCs arrest in capillary beds, their prolonged residence increases sustained contact with platelets, neutrophils, and endothelial cells and drives acquisition of an immunosuppressive phenotype. Platelets adhering directly to MTCs induce CD155 expression and suppress NK cell degranulation and IFN‐γ production through the CD155–TIGIT axis [118]. Platelet‐derived RGS18 is taken up by MTCs and activates AKT–GSK3β–CREB signaling to upregulate HLA‐E, strengthening the inhibition of NKG2A^+^ NK cells [16]. Tumor‐induced PD‐L1^+^ reticulated platelets can confer an apparent PD‐L1‐positive phenotype on T cells, NK cells, and other immune cells, further intensifying systemic immunosuppression [178]. Thrombin also induces ectodomain shedding of the activating NK cell ligand Necl5 from MTCs, protecting cells arrested in pulmonary capillaries from NK‐mediated killing [179]. Platelet‐derived chemokines recruit neutrophils and generate MTC–platelet–neutrophil complexes that inhibit the intraluminal NK cell clearance of arrested cancer cells and facilitate extravasation [180]. Heterotypic MTC–neutrophil clusters also drive MTC cell cycle entry and increase metastatic seeding efficiency [116]. In CRC, NNMT‐high pericytes activate endothelium and retain neutrophils through CXCL5–CXCR2 signaling, allowing MTC–neutrophil clusters to form at the vascular‐immune interface of the primary tumor [181]. Activated neutrophils further release NETs that capture MTCs and spatially separate them from cytotoxic T lymphocytes and NK cells. NET‐derived DNA is recognized by CCDC25 on MTCs, inducing chemotaxis and distant colonization [182, 183, 184]. Tumor‐derived autophagosomes induce PD‐L1‐bearing NETs through HMGB1–TLR4–ERK/p38 signaling, combining a physical barrier with checkpoint‐mediated immunosuppression [185].

CTM formation in the circulation actively regulates both immunity and proliferation. Cancer cell PLXNB2 binds monocyte SEMA4A to promote homotypic MTC aggregation and heterotypic MTC–monocyte clustering [186]. Exhausted, immunosuppressive CD4^+^ CD8^+^ double‐positive T cells form heterotypic clusters with MTCs through VLA‐4‐VCAM1 and enhance pulmonary seeding [187]. A ROS–Notch–Nodal positive‐feedback circuit between polymorphonuclear myeloid‐derived suppressor cells (MDSCs) and MTCs further maintains MTC survival, proliferation, and stemness [188]. Together, these mechanisms enable MTCs to evade dual surveillance by NK and T cells, survive in the circulation, and seed distant organs.

#### 4.4.2 Immune Regulation Within the Lymphatic mTME

LNs are both critical sites for initiating antimetastatic immunity and major hubs through which tumors establish local and systemic immune tolerance. Current evidence suggests that nodal immunity follows a continuum from immune surveillance through immune remodeling and tolerance to eventual immune collapse.

LN colonization is accompanied by a systemic transition in immune state. Early tdLNs can support tumor‐specific T cell responses, while antitumor immunity and immune reservoir function progressively deteriorate as nodal metastasis advances [189]. During immune surveillance, tdLNs retain their antitumor reservoir function, and recirculating immune populations impose antimetastatic pressure [190]. CD169^+^ subcapsular sinus macrophages capture lymph‐borne tumor EVs and restrict their access to the nodal cortex, providing a physical barrier that gradually weakens with tumor progression [191]. Concurrently, cDC1s within tdLNs preserve relatively stable TCF1^+^PD‐1^+^ tumor‐specific progenitor exhausted T cells (Tpex), prevent progressive dysfunction, and help replenish intratumoral effector T cells [192, 193].

With metastatic progression, however, immune remodeling can begin in LNs before MTC colonization. Tumor‐derived soluble factors induce fibroblastic reticular cell (FRC) proliferation and reticular‐network remodeling in tdLNs, downregulate CCL21 and IL‐7, and alter immune cell recruitment, localization, and survival [194]. In HNSCC, tumor‐derived EVs carrying IFNGR1 also induce FRC PD‐L1 expression through JAK1–STAT1 signaling, suppressing CD8^+^ T cells and establishing the PMN [195]. FRC activation may therefore precede detectable Treg expansion and disruption of Tpex responses and, together with the deterioration of the subcapsular–sinus barrier and recruitment of suppressive myeloid cells, constitutes an early transition from immune surveillance to immunoediting.

After cancer cells enter LNs, stromal preconditioning becomes coupled to Treg and myeloid‐mediated suppression. An MTC‐intrinsic interferon response program promotes NK cell evasion, while LN colonization further induces Tregs and thereby establishes the systemic immune tolerance that facilitates the colonization of distant organs [122]. Activated nodal Tregs also compete for IL‐2 and restrict granzyme B and perforin expression in tumor‐specific CD8^+^ T cells, reducing cytotoxicity. This finding indicates that the physiological immune permissiveness of LNs is amplified after tumor colonization [196]. Therapy‐generated tumor debris can be phagocytosed by medullary sinus macrophages, triggering the IL‐33/ST2 signaling that activates Tregs in tdLNs and promotes their migration to tumors, providing an additional route to treatment‐associated immune tolerance [197].

As metastatic burden increases, cumulative immune remodeling progressively culminates in nodal immune collapse. In uninvolved LNs, anti‐PD‐L1 therapy activates Tpex near dendritic cells and promotes their differentiation into proliferative intermediate exhausted T cells (Tex‐int). In metastatic LNs, by contrast, Tpex and Tex‐int cells are confined to suppressive niches enriched in proliferating Tregs and regulatory dendritic cells and cannot mount an effective treatment response [120]. Immune collapse is further accompanied by the replacement of nodal architecture with CAF‐myeloid suppressive niches. Spatial multiomics showed that niches formed by immunosuppressive myeloid cells and CAFs disrupt perifollicular T cell zones and lymphoid follicles and are associated with T cell dysfunction, Treg activation, and immune alterations in adjacent uninvolved LNs [122]. SPP1^+^ tumor‐associated macrophages (TAMs) may activate fibroblasts and promote T cell exhaustion through SPP1–CD44 and CD155–CD226 interactions, creating a local niche that supports expansion and colonization of CXCR4^+^MTCs [198]. In patients with CRC liver metastasis, metastatic tdLNs contain fewer T and B cells but more fibroblasts, endothelial cells, and macrophages, indicating the conversion from a lymphocyte‐dominant immune organ into a stromal and myeloid dominant metastatic niche [199]. Cancer cells also acquire mitochondria from antigen‐presenting cells, CD8^+^ T cells, and NK cells after direct contact, simultaneously impairing donor immune function and activating MTC cGAS–STING–Type I interferon signaling to further amplify immune collapse [200]. These findings suggest that, from late immune surveillance to early immunoediting, PD‐L1 blockade directed to tdLNs, restoration of Tpex states, and relief of Treg‐mediated suppression may delay immune remodeling. Once myeloid‐stromal replacement of LN architecture has occurred, checkpoint blockade alone is unlikely to restore antitumor immunity; the combined targeting of stromal, myeloid, and Treg networks may be required. The sequential remodeling of lymphatic vessels and tdLNs, together with the reciprocal interactions among MTCs, stromal cells, and immune cells, is summarized in Figure 4.

**FIGURE 4:**
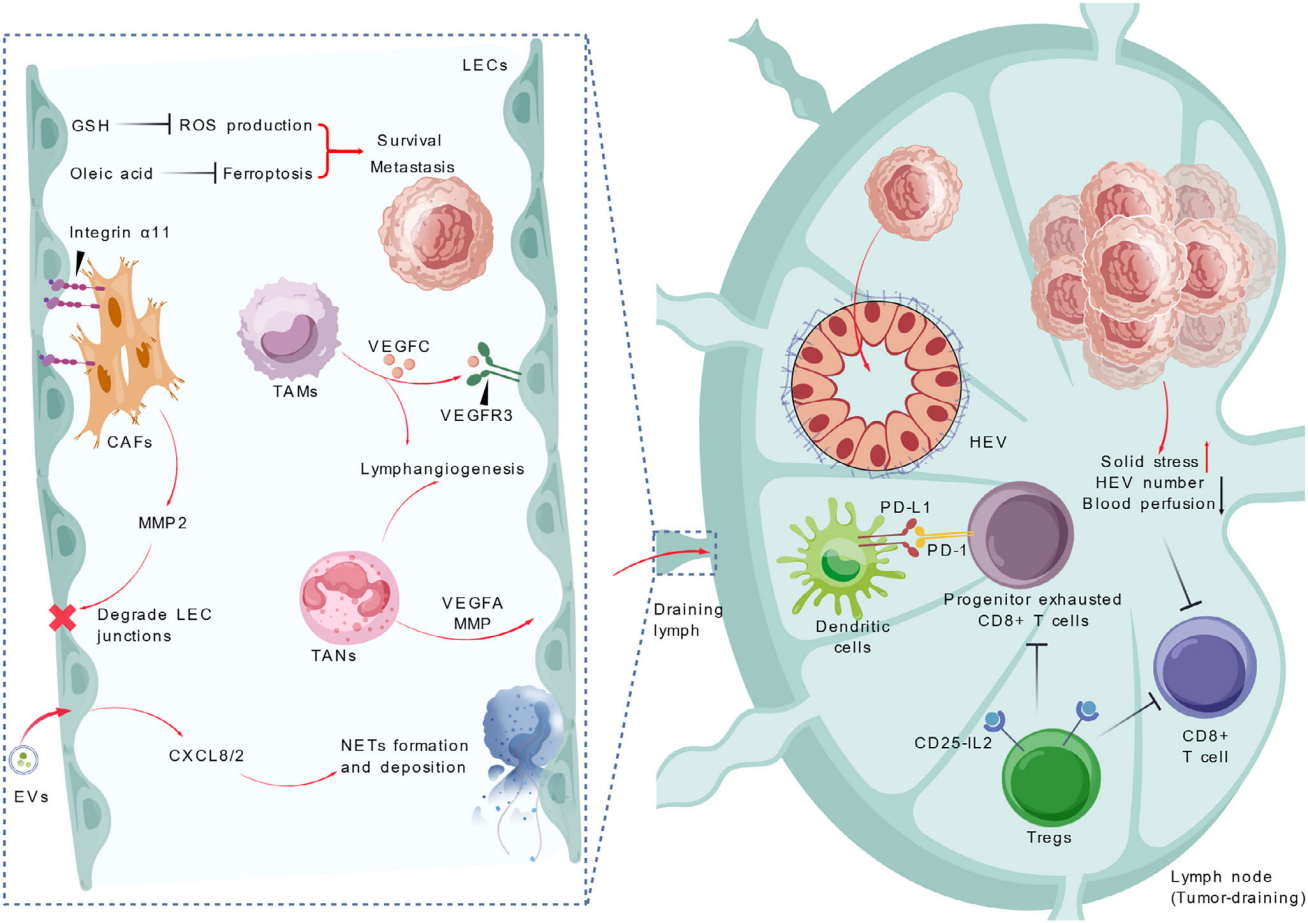
Schematic illustrating reciprocal interactions between MTC–lymphatic mTME. MTCs undergo metabolic reprogramming within lymphatic vessels to enhance survival before entering hematogenous circulation via HEVs. TAMs, TANs, and CAFs promote lymphatic metastasis by degrading LEC junctions, promoting lymphangiogenesis, and depositing NETs. Within tdLNs, immune suppressive microenvironment is established through stromal remodeling, expression of immune checkpoints, and interactions between effector T cells, Tregs, and dendritic cells. Abbreviations: CAF, cancer‐associated fibroblast; CXCL8/2, C–X–C motif 8/2; GSH, glutathione; EV, extracellular vesicle; HEV, high endothelial venule; LEC, lymphatic endothelial cell; MMP, matrix metalloproteinase; PD‐1, programmed death‐1; PD‐L1, programmed cell death ligand 1; TAM, tumor‐associated macrophage; TAN, tumor‐associated neutrophil; VEGFC, vascular endothelial growth factor C. Created with BioGDP.com.

#### 4.4.3 Immune Regulation Within the Perineural mTME

Subcellular‐resolution spatial transcriptomics has shown that PNI regions are enriched in SCs, M2‐like macrophages, CAFs, and B/plasma cells, with a concomitant reduction in exhausted CD8^+^ T cells. Stromal CAF–macrophage spatial clusters jointly express fibrotic and inflammatory programs, whereas cancer cells activate ECM remodeling and axonogenesis pathways [201]. Immune regulation during perineural migration should therefore be understood as a process jointly driven by nerve injury, glial repair, and myeloid and stromal remodeling. On the basis of current evidence, PNI can be organized into three overlapping functional stages: the establishment of neurotropism, adhesion to and disruption of the perineurial barrier, and intraneural dissemination. Immune regulation and remodeling actively contribute throughout all three stages.

During the establishment of neurotropism, before MTCs invade nerves, neuropeptides, neurotrophic factors, tumor‐derived soluble mediators, and EVs can already remodel the immune milieu. In PDAC, nociceptor‐derived CGRP acts on RAMP1 in CAFs and suppresses IL‐15 expression, reducing NK cell infiltration, proliferation, and cytotoxicity; CAF‐derived NGF reciprocally activates sensory nerves, forming an NGF–CGRP positive feedback loop [129]. Cholangiocarcinoma‐derived exosomal LINC01812 induces M2‐like macrophage polarization and neurotrophic‐factor release through TUBB4B–Notch–CCL2 signaling, enhancing MTC migration toward DRG [202]. In addition, MTC‐derived CSF‐1 recruits endoneurial macrophages that secrete GDNF and promote neurotropic MTC migration through GFRA1–RET–ERK signaling [203]. This stage is therefore characterized by reduced NK cell pressure and the recruitment of protumor myeloid cells that reinforce neurotropism.

SCs may be the critical intermediary linking neurotropic signals to physical invasion. In cervical cancer, cancer‐derived neuromedin B activates NMBR–PKA–Ca^2+^ signaling in SCs, inducing their proliferation, migration, CCL2 secretion, and axonal regeneration and creating a nerve‐tumor bridge [154]. Tumor‐activated SCs then initiate a c‐JUN‐dependent repair program and form dynamic migratory tracks resembling bands of Büngner [131]. Upon direct contact, GFAP^+^NCAM1^+^ SCs induce polarized protrusions toward MTCs, intercalate between them, disrupt intercellular junctions, and guide single‐cell migration [155]. SCs therefore do not merely respond to the established PNI but can actively initiate neural invasion. During adhesion to and disruption of the perineurial barrier, SC‐derived CCL2 selectively recruits CCR2^+^ inflammatory monocytes to PNI sites and induces monocyte differentiation into cathepsin B^+^ macrophages, which degrade Collagen IV and other matrix components of the perineurium and basement membrane [128]. Concurrently, MTC ITGA5 binds FN1 in the SC matrix, further inducing repair‐like SCs and NGF release, promoting neurogenesis while suppressing NK cell infiltration and IFN‐γ‐dependent killing [204]. Loss of perineurial and myelin integrity may therefore mark the transition from relatively reversible neurotropism to adhesive neural invasion and the formation of an immunosuppressive niche.

Once cancer cells enter nerve fascicles, persistent neural injury further drives local immunosuppression. A temporal study of PNI showed that MTCs degrade myelin and induce neuronal ATF3/JUN injury programs. As the burden of invaded nerves increases, neuron‐derived IL‐6 and Type I interferon responses become chronically activated, accompanied by the accumulation of CD163^+^PD‐L1^+^ macrophages, NETs, and TIM3^+^ or LAG3^+^ exhausted CD8^+^ T cells, ultimately producing resistance to anti‐PD‐1 therapy [127]. Intraneural dissemination also involves glial‐myeloid positive feedback and neurotransmitter‐mediated suppression of adaptive immunity. TAM‐derived basic fibroblast growth factor activates SCs through PI3K–AKT. SCs then release IL‐33, recruit additional macrophages, and promote M2‐like polarization, establishing a reciprocal circuit that sustains PNI [205]. Severe PNI also increases acetylcholine, which suppresses cancer cell CCL5 through HDAC1, reduces CD8^+^ T cell recruitment, and directly inhibits CD8^+^ T cell IFN‐γ production and T helper 1 differentiation [206]. A transiently protective nerve injury repair response is thereby converted into a persistent state that remodels tumor‐wide immunity, characterized by dominant myeloid suppression, impaired NK cell function, and T cell exhaustion.

## 5 Metastatic Niche

Upon reaching distant organs and entering the target organ parenchyma, cancer cells may diverge toward immune elimination, transient retention, single‐cell dormancy, regression of nascent micrometastases, or immediate outgrowth. Importantly, not all cancer cells traverse the same states or follow an identical sequence. Their fate is determined by the degree of compatibility between the plasticity, stress tolerance, and immune‐evasive capacity of metastasis‐initiating cells and the organ‐resident cells, ECM, metabolic milieu, and local and systemic immune states of the target tissue [1, 207].

At the spatial level, the dimensions of transit routes within the target organ and the positioning of MTCs relative to organ microvessels, vascular branch points, and capillary basement membranes are critical determinants of mechanical arrest and subsequent extravasation [208]. Temporally, PMN formation can precede the arrival of MTCs. EVs, cytokines, chemokines, and metabolites released by the primary tumor enter the circulation and reprogram distant endothelial cells, fibroblasts, the ECM, and both organ‐resident and bone marrow‐derived immune cells, thereby establishing a PMN. Following MTC arrival, reciprocal interactions with this preconditioned milieu further transform the PMN into an early metastatic niche that supports adhesion, extravasation, immune evasion, dormancy, or metastatic outgrowth [209, 210].

The metastatic niche therefore exhibits both temporal continuity and spatial hierarchy. Because distinct cancer types, target organs, and metastatic stages engage different niche components and interaction programs, MTC behavior within distant organs should be interpreted within the broader ecological context of the metastatic niche. Representative mechanisms governing MTC adhesion to organ‐specific endothelium and subsequent extravasation into the liver, lung, and brain are summarized in Figure 5.

**FIGURE 5:**
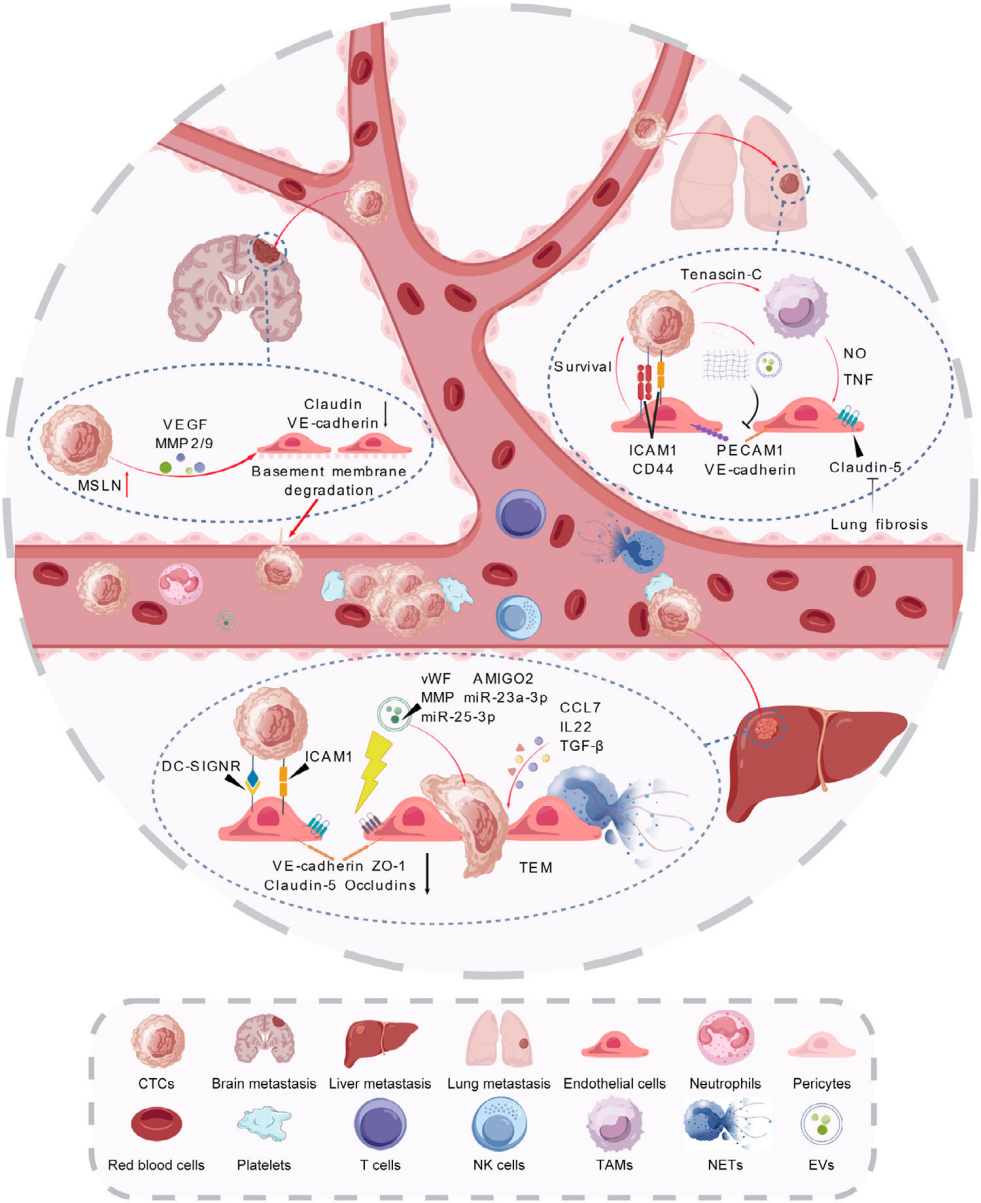
Schematic representation of metastatic niche. This figure displays representative mechanisms of establishment of metastatic niche. Soluble factors and extracellular vesicles can be transmitted to metastatic site (liver, lung, and brain) and induce PMN formation by disrupting endothelial junctions and basement membrane degradation. Upon MTC arrival, the permissive PMN facilitates their vascular endothelium and subsequent extravasation into the parenchyma. Within parenchyma, MTCs actively undergo metabolic reprogramming and interact with tissue‐resident cells to reinforce colonization and exacerbate metastatic burden. Abbreviations: AMIGO2, amphoterin‐inducible gene and open reading frame 2; ICAM1, intercellular adhesion molecule 1; IL‐22, interleukin‐22; MMP, matrix metalloproteinases; MSLN, mesothelin; NO, nitric oxide; PECAM1, platelet endothelial cell adhesion molecule 1; TEM, transendothelial migration; TNF, tumor necrosis factor; VEGF, vascular endothelial growth factor; vWF, von Willebrand Factor; ZO‐1, zonula occludens‐1. Created with BioGDP.com.

### 5.1 Liver Metastatic Niche

Portal venous drainage gives cancer cells preferential access to the liver, whereas slow sinusoidal blood flow prolongs MTC arrest. Liver sinusoidal endothelial cells lack a continuous basement membrane, and hepatic stellate cells in the abluminal space of Disse can likewise be reprogrammed to induce PMN formation and sustain a metastasis‐supportive milieu. Moreover, hepatic immune homeostasis maintained by Kupffer cells and NK/NKT cells can be hijacked to establish immune tolerance. Thus, the physiological architecture and resident cellular composition of the liver jointly provide a stable, permissive environment for metastatic colonization [211].

Before MTC arrival, EVs can remotely establish a metastasis‐permissive state in the liver. EV‐delivered miRNAs and membrane proteins remodel the hepatic microvascular endothelial barrier, increase vascular permeability, and induce a PMN that facilitates MTC extravasation [212, 213, 214]. Beyond increasing vascular leakage, EVs also reprogram the adhesive phenotype of liver sinusoidal endothelium. EVs released by AMIGO2^+^ gastric cancer cells transfer AMIGO2 to liver sinusoidal endothelial cells, thereby enhancing their adhesion to MTCs [215]. Kupffer cells also selectively take up EVs bearing surface integrin αvβ5, promoting liver‐tropic metastasis [216]. EVs can further coordinate the reprogramming of Kupffer cells, hepatic stellate cells, MDSCs, and NK cells, reshaping the hepatic immune niche before MTC arrival. MIF carried by PDAC‐derived exosomes induces Kupffer cell secretion of TGF‐β, activates hepatic stellate cells, increases fibronectin deposition, and recruits bone marrow‐derived macrophages [217]. In CRC‐derived EVs, miR‐21‐5p and CDC42 induce prometastatic inflammation through TLR7–IL‐6 and NOD1 signaling, respectively [218, 219]. As the metastatic niche evolves, EV‐derived miR‐934 promotes protumor macrophage polarization through the PTEN–PI3K–AKT axis; TGF‐β1 carried by CRC‐derived EVs converts hepatic stellate cells toward a CAF‐like phenotype, activates CXCL12 signaling to recruit MDSCs, and downregulates NKG2D on NK cells to attenuate cytotoxicity [220, 221]. Collectively, the EV‐driven hepatic PMN integrates endothelial barrier disruption, enhanced sinusoidal adhesion, immune cell reprogramming, and impaired antitumor immunity, thereby establishing a permissive scaffold for MTCs to form macrometastases.

During MTC extravasation, strengthened tumor–endothelial adhesion and progressive endothelial‐barrier disruption accelerate transendothelial migration. Fibronectin deposits on the luminal surface of hepatic vessels provide Talin‐1/integrin‐binding sites for MTCs, stabilizing adhesion and membrane protrusions [222]. Cancer cells actively secrete IL‐23 and related signals, inducing autocrine TNF‐α production by liver sinusoidal endothelial cells; this response accelerates F‐actin depolymerization, upregulates MMP9 and ICAM1, and generates pathologically enlarged intercellular gaps that facilitate transendothelial passage [223]. In a mouse model of CRC liver metastasis, MTC stimulation prompted liver‐resident iNKT17 cells to release IL‐22, which upregulated endothelial ANPEP and further increased endothelial permeability [224].

After entering the hepatic parenchyma, MTCs may undergo immune clearance, enter dormancy, or expand. Hepatic NK cells can induce MTC dormancy through IFN‐γ; upon activation, however, hepatic stellate cells drive NK cell quiescence via CXCL12–CXCR4 signaling, allowing dormant cells to resume proliferation [225]. As micrometastases expand, an immunosuppressive liver metastatic microenvironment progressively emerges. Macrophage‐derived progranulin and MTC‐derived leukemia inhibitory factor cooperatively activate STAT3 signaling in metastasis‐associated fibroblasts, promoting periostin secretion, inducing an immunosuppressive macrophage phenotype, and suppressing cytotoxic T cell function [226]. In human CRC liver metastases, SPP1^+^ macrophages and fibroblasts form a spatially juxtaposed immunosuppressive network associated with poor prognosis [227]. Concurrently, TGF‐β impedes the recruitment of circulating memory CD8^+^ T cells and induces SPP1 expression in macrophages, suppressing clonal expansion of newly infiltrating T cells and establishing a dual barrier characterized by both immune exclusion and functional exhaustion [228]. In established liver metastases, FasL^+^ monocyte‐derived macrophages induce apoptosis of activated Fas^+^ CD8^+^ T cells, impairing both intrahepatic and systemic immunity and reducing responses to immune‐checkpoint blockade [229].

Single‐cell and multiomics analyses have further resolved the spatiotemporal evolution of the liver metastatic microenvironment. During PMN formation, hepatocytes activate IL‐6/JAK/STAT3 and TNF‐α/NF‐κB signaling and express inflammatory and myeloid recruiting factors, reinforcing the inflammatory PMN phenotype [230]. Through IL‐6–STAT3 signaling, tumor‐associated inflammation reduces intratumoral infiltration by dendritic cells and T cells and induces hepatocyte production of LRG1, which promotes the accumulation of immature, MDSC‐like neutrophils and compromises effector T cell and dendritic cell function [231, 232]. During metastatic progression, immunosuppressive neutrophil subsets, Tregs, and exhausted CD8^+^ T cells progressively accumulate. Concurrent PD‐L1 upregulation in hepatocytes, neutrophils, and macrophages further accelerates CD8^+^ T cell exhaustion [230]. Once metastases are established, the enrichment and interaction of exhausted T cells, activated Tregs, suppressive macrophages, and fibroblasts form the cellular basis of local immunosuppression [233]. Exhausted T cells are more frequent within profibrotic, α‐SMA‐rich regions of liver metastases and interact more closely with SPP1^+^ macrophages [234]. Paired single‐cell profiling and spatial imaging further demonstrated complementary ligand–receptor networks and spatial proximity between SPP1^+^ macrophages and fibroblasts in CRC liver metastases; together, these populations remodel the ECM and induce immunosuppression [227].

Microbial signals further remodel the liver metastatic habitat through the gut–liver axis. Multiple studies suggest that liver metastasis is frequently accompanied by disruption of the gut vascular barrier, permitting intestinal bacteria to reach the liver through the portal circulation. Microbiota‐driven recruitment of neutrophils and macrophages increases hepatic inflammation and promotes adaptive remodeling of the PMN [100]. In a preclinical hepatocellular carcinoma model, gut dysbiosis enhanced NETosis and VEGF‐driven angiogenesis, thereby facilitating hepatic metastatic colonization [235]. Conversely, pharmacological intervention altered the functional composition of the microbiome, reduced infiltration by prometastatic CCR2^+^ Ly6C^+^ monocytes, and enhanced T helper 1 and CD8^+^ T cell effector function, collectively restraining metastatic progression [236].

Overall, formation of the liver metastatic niche proceeds as a stepwise program of immune escape. Tumor‐derived EVs remotely disrupt the sinusoidal endothelial barrier, increasing vascular permeability and inducing immunosuppression. MTCs then complete extravasation through adhesive interactions and tumor‐induced widening of endothelial junctions. Within metastatic lesions, spatially organized networks formed by MTCs, macrophages, and fibroblasts promote T cell exclusion and exhaustion; these networks are consolidated by cascading myeloid suppression and stromal interactions and further reinforced by microbial translocation and host–microbiome crosstalk. The resulting niche couples local immune escape with systemic immune dysfunction.

### 5.2 Lung Metastatic Niche

The lungs receive the entire systemic venous return and contain a dense capillary bed; they have also evolved an exceptionally thin alveolar–capillary barrier to support efficient gas exchange. These features directly favor intravascular arrest and extravasation of MTCs [237]. Nevertheless, the continuous pulmonary capillary endothelium excludes most MTCs. In an experimental melanoma model, approximately 70% of cancer cells entering the lungs were cleared within 6 h, illustrating the high arrival but low productive colonization phenotype of pulmonary metastasis [238]. Remodeling of the pulmonary niche is therefore a prerequisite for successful metastatic colonization.

Before MTC arrival, primary tumors can remotely induce a pulmonary PMN through soluble factors, EVs, and systemic inflammation. In a mouse model of breast cancer lung metastasis, tumor‐derived factors activated pulmonary fibroblasts, promoting collagen remodeling and MMP expression and increasing matrix stiffness and vascular leakage [239]. At the vascular level, tumor‐derived Angiopoietin‐2 suppresses Claudin‐5 in pulmonary endothelial cells through FOXO1, disrupting cellular tight junction [240]. In parallel, α2,6‐sialylated EVs trigger endoplasmic–reticulum stress through PERK/eIF2α signaling, disrupt intercellular junctions and increase pulmonary vascular permeability [241].

Immune remodeling of the pulmonary PMN is characterized by cooperation between tissue‐intrinsic stromal programs and remote signals from the primary tumor. Under homeostatic conditions, pulmonary adventitial fibroblasts impair dendritic cell function and suppress monocytes through COX‐2–PGE2 signaling; this program is amplified by primary tumor‐derived IL‐1β and propagated by tumor‐associated inflammation [242]. Breast cancer cell‐released autophagosomes activate TLR4–MyD88–ERK/p38 signaling and induce PAD4‐dependent NET formation. PD‐L1 displayed on these NETs suppresses CD8^+^ T cell proliferation and IFN‐γ production, thereby reducing cytotoxic activity [185]. EVs from Lin28B‐high breast cancer cells likewise recruit neutrophils, upregulate PD‐L2, and establish an immunosuppressive state [243].

Beyond structural and immune remodeling, the pulmonary PMN undergoes metabolic preconditioning. Primary tumors increase pulmonary aspartate levels, which activates NMDAR–CREB–DOHH signaling to enhance production of TGF‐β‐pathway proteins and collagen synthesis, allowing a nutrient to function simultaneously as a signaling molecule and a niche‐remodeling factor [244]. PMN formation also selectively enriches pulmonary palmitate, enabling MTCs to sustain prometastatic NF‐κB signaling [245]. In addition, IL‐1β induces lipid accumulation in pulmonary mesenchymal cells, which transfer lipids to MTCs and NK cells, thereby promoting MTC survival while suppressing NK cell function [246].

After MTCs reach pulmonary capillaries, their arrest, adhesion, and transendothelial migration are jointly governed by hemodynamics, endothelial state, and cell adhesion capacity. The Cdc42 effectors IQGAP1 and N‐WASP maintain β1‐integrin expression through ERK/FAK signaling and enhance MTC adhesion to pulmonary endothelium under shear stress through MRTF–SRF signaling [247]. NET‐derived DNA also serves as a chemotactic ligand recognized by CCDC25 on MTCs, activating the ILK‐β–parvin pathway to promote directional migration [183]. After stable adhesion has formed, hypoxia‐induced PLOD2 in MTCs modifies Collagen VI, which engages ITGβ1 on pulmonary endothelial cells, promotes its colocalization with Kindlin‐2 and interaction with F‐actin, and drives F‐actin polymerization, VE‐cadherin internalization, and junctional disruption [248]. Primary tumor‐derived proangiogenic factors can also downregulate endothelial TRAIL, relieving intracrine inhibition of DR5 signaling and thereby inducing endothelial cell death, NF‐κB/p38 activation, and upregulation of ICAM‐1 and E‐selectin. These changes increase myeloid cell infiltration, MTC adhesion, and vascular permeability [249].

Once MTCs enter the pulmonary parenchyma, they can switch between dormancy and proliferative growth. Single‐cell tracking showed that some cells proliferate intravascularly, whereas others extravasate in response to Wnt signals from pulmonary endothelium and enter a relatively stable latent state [250]. During this interval, alveolar macrophages maintain breast cancer cell dormancy through TGF‐β2–TGFBR3 signaling. Aggressive cells evade this restraint by reducing cell–cell contact or downregulating TGFBRIII [251]. An MTC‐intrinsic ZFP281 program maintains a mesenchymal‐like, slowly proliferating dormant state, whereas loss of ZFP281 promotes an epithelial‐like proliferative phenotype and macroscopic lung metastasis [252]. Reactivation of dormant cells is jointly regulated by ECM remodeling and changes in host state. In mouse models, for example, NETs induced by chronic inflammation use elastase and MMP9 to cleave laminin, expose cryptic matrix epitopes, and activate MTC FAK/ERK/YAP signaling, thereby awakening dormant cells [253].

The immune milieu of lung metastasis subsequently shifts from early inflammation to spatially organized immunosuppression. Time‐resolved single‐cell and spatial analyses showed that inflammatory neutrophils and monocytes appear first during PMN formation, followed by progressive accumulation of suppressive macrophages as micrometastases emerge. TREM2^+^ regulatory macrophages selectively accumulate at invasive margins and form an immunosuppressive boundary that restricts immune cell access to the tumor [254]. At the macrometastatic stage, metastatic lesions increasingly reprogram lung‐resident cells. Through their secretome, breast cancer lung metastases activate SREBP1 in adjacent alveolar Type II cells, upregulate FASN and GPAM, and convert these cells into lipid donors that sustain metastatic growth [255]. Therapy can also reshape the niche; for example, doxorubicin induces complement production by pulmonary fibroblasts, recruits MDSCs, and suppresses T cell function [256].

In summary, the pulmonary metastatic microenvironment is progressively reshaped by anatomical susceptibility, disruption of homeostatic barriers, immune and metabolic adaptation, and reciprocal remodeling by macrometastases into an ecosystem that supports metastatic colonization. Pulmonary endothelial cells, fibroblasts, alveolar macrophages, and neutrophils may exert either antimetastatic or prometastatic effects at different stages, underscoring marked spatiotemporal heterogeneity. Effective targeting of the pulmonary metastatic niche must therefore account for both the cellular state of the metastatic lesion and the timing of intervention.

### 5.3 Brain Metastatic Niche

The BBB comprises brain microvascular endothelial cells with continuous tight junctions and low transcytotic activity, together with pericytes, basement membrane, and astrocytic endfeet; these components interact with neurons and microglia to form the neurovascular unit. Beyond restricting the entry of cells and macromolecules, brain endothelium maintains ionic and metabolic homeostasis in the parenchyma through selective transport, active efflux, and low basal leukocyte trafficking. Cerebral microvessels therefore constitute both the principal anatomical barrier to MTC colonization and, in response to tumor‐derived signals, a vascular niche that regulates MTC fates. Before MTCs arrival and during early extravasation, they encounter a relatively intact BBB; as metastases enlarge, local vessels progressively evolve into a blood–tumor barrier (BTB). Although the BTB often exhibits abnormal junctions and increased permeability, efflux transport, and pericyte coverage vary markedly both between and within lesions; consequently, anticancer drugs do not necessarily penetrate brain metastases uniformly [257].

Before MTC arrival, primary tumors may remotely modulate the cerebrovascular interface through EVs, migrasomes, and soluble factors. Even without direct parenchymal invasion, primary‐tumor‐associated inflammatory stimuli can activate brain endothelial cells and alter endothelial junctions, adhesion‐molecule expression, and immune cell trafficking [258]. Preclinical models indicate that ATF6‐enriched migrasomes released by breast cancer cells are taken up by brain endothelial cells, inducing endoplasmic–reticulum stress and disrupting intercellular junctions [259]. NSCLC‐derived exosomes deliver lnc‐MMP2‐2, which downregulates tight junctions and adherens junction proteins through the miR‐1207‐5p/EPB41L5 axis [260]. Hypoxia also increases production of ITGB3‐containing EVs. After retention within brain microvascular endothelial cells, these EVs enhance VEGFR2 signaling, increase endothelial permeability, and promote MTC transendothelial migration [261].

As MTCs reach cerebral capillaries, their arrest and extravasation are jointly determined by hemodynamics, adhesion molecules, endothelial paracrine signals, and local barrier remodeling. In an in vitro selection model, breast cancer subpopulations with high affinity for brain endothelium were enriched for MUC1, VCAM1, and VLA‐4 and displayed enhanced transendothelial migration and survival on soft, brain‐mimetic matrices [262]. Soluble factors released by brain endothelial cells further activate EGFR–DOCK4–RAC1 signaling in cancer cells, inducing MTC intercalation into brain endothelium and promoting capillary arrest [263]. MTCs arrested within cerebral capillaries release MMP9, inducing luminal endothelial projections and pocket‐like vascular structures that create a permissive interface for extravasation [264]. Occlusion of microvessels by MTCs can also slow local blood flow and generate a microinfarct‐like hypoxic milieu. This process induces endothelial Angiopoietin‐2 and cancer cell VEGF, destabilizes the BBB, and establishes an ischemia‐like niche favorable for colonization [265]. Notably, MTCs can acquire an extravasation advantage without a pronounced global increase in BBB permeability. In a three‐dimensional BBB model, astrocyte‐derived CCL2 acted on CCR2‐expressing cancer cells, directing their migration toward astrocyte‐rich regions, facilitating transit across the brain microvasculature, and increasing capillary arrest and extravasation [266]. BBB remodeling in brain metastasis should therefore be viewed as a dynamic process encompassing changes in permeability, endothelial remodeling, and chemotactic MTC migration rather than simply disruption of intercellular junctions.

After entering the brain parenchyma, MTCs initially depend on the perivascular niche for survival and subsequently transition among dormancy, death, and proliferative growth. Laminin‐211 derived from astrocytic endfeet binds MTC dystroglycan, promotes cytoplasmic retention of YAP, and maintains dormancy of breast cancer MTCs [267]. Dormant cells are still metabolically active. Dormant brain metastatic cells use DRP1 to sustain FAO, energy production, and redox homeostasis, thereby acquiring the capacity for long‐term survival and subsequent reactivation [268]. Reprogrammed by MTCs, astrocytes reciprocally promote MTC immune evasion. Reprogrammed astrocytes induce MTC CDK5, suppress NLRC5 and MHC‐I expression and antigen presentation, and enable evasion of CD8^+^ T cell recognition [269]. pSTAT3^+^ astrocytes also secrete TIMP1, which acts on CD63^+^CD8^+^ T cells to establish local immunosuppression [270].

Overall, the brain metastatic niche arises through coordinated remodeling among cancer cells, the cerebral vasculature, and neural components. Cancer cells enter the brain through vascular adhesion, focal BBB disruption, and transendothelial migration and subsequently reprogram endothelial cells, pericytes, and glial cells. These interactions establish a perivascular niche characterized by vascular abnormalities, hypoxia, matrix remodeling, and immune dysregulation, ultimately supporting the survival, colonization, and growth of metastatic cells.

## 6 Research Models for Metastasis and Multiomics Applications

Metastasis is a dynamic process encompassing local invasion, survival in the circulation, vascular arrest, extravasation, organ homing, dormancy, and colonization. Metastasis research therefore requires stage‐matched, multiscale models.

Conventional 2D culture systems are tractable and readily amenable to genetic or pharmacological perturbation, but fail to reproduce FSS, dynamic nutrient gradients, vascular barriers, and multicellular interactions. Tumor‐on‐chip and metastasis‐on‐chip platforms integrate microfluidics, 3D culture, and tissue engineering to model perfusion, tumor ECM, abnormal vasculature, and immune interactions under controlled conditions. These systems provide versatile platforms for studying tumor evolution, metastatic mechanisms, drug screening, and precision therapy [271]. For example, a breast cancer metastasis‐on‐chip model integrating MDA‐MB‐231 cells, fibroblasts, and perfusable microvessels demonstrated that tumor and fibroblast coculture disrupts the endothelial barrier and enhances cancer cell invasion and intravasation [272]. Combining patient‐derived organoids with organ‐on‐chip technology further improves clinical relevance. PDAC‐derived organoids cultured with pancreatic stellate cells and macrophage‐like cells recreate key features of a fibrotic and immunosuppressive TME and show that stromal targeting can enhance gemcitabine‐induced cancer cell apoptosis [273]. Such models are particularly useful for defining the contributions of CAFs and TAMs to invasion, drug resistance, and metastatic adaptation.

Nevertheless, in vivo models remain indispensable for validating metastatic competence and treatment responses. Zebrafish embryos are transparent, readily imaged, and amenable to high‐throughput analysis, allowing real‐time visualization of intravasation, vascular arrest, and extravasation [274]. They are therefore well suited to studying early hematogenous dissemination and single‐cell behavior. Orthotopic patient‐derived xenograft (PDX) models preserve the genetic background and intratumoral heterogeneity of patient tumors while recreating the relevant primary organ microenvironment and spontaneous dissemination, making them among the most clinically relevant in vivo models for metastasis research. In CRC, cells capable of forming orthotopic PDX tumors and metastases exhibit stronger EMT and TGF‐β pathway activation, increased MTC and CD44^+^ cluster formation, and greater drug resistance [275]. These findings highlight the influence of the in vivo microenvironment on metastatic behavior. However, conventional PDX models rely on immunodeficient hosts, and human stromal and immune components are progressively replaced by murine cells. They are therefore unsuitable for directly modeling the complete human antitumor immune response.

Metastasis research is increasingly shifting from static, mutation‐centered maps toward multiomics frameworks integrating genomic, transcriptomic, single‐cell lineage, and spatial information. The AURORA US study showed that most paired primary tumors and metastases retained substantial molecular similarity [276]. Similarly, Cas9‐based single‐cell lineage tracing in PDX models demonstrated that differences in clonal metastatic competence arise from cellular states that pre‐exist transplantation, suggesting that metastatic progression does not uniformly require de novo metastasis driver mutations [20]. Instead, DNA methylation‐mediated repression of cell adhesion genes, epigenetic silencing of *HLA‐A*, and reduced immune infiltration can jointly promote the invasiveness and immune evasion of metastatic lesions. Metastasis should thus be viewed as a dynamic adaptive process shaped by genetic background, epigenetic reprogramming, and microenvironmental selection pressures.

Single‐cell and spatial multiomics approaches further resolve the spatial heterogeneity of metastatic lesions. The integration of single‐cell RNA sequencing (scRNA‐seq) or single‐nucleus RNA sequencing (snRNA‐seq) with Slide‐seq, multiplexed error‐robust fluorescence in situ hybridization, expansion sequencing, codetection by indexing (CODEX), and histopathological imaging has revealed spatially distinct malignant, macrophage, T/NK cell, and stromal cell states in metastatic breast cancer, including EMT‐low, EMT‐patched, and EMT‐high cancer cell phenotypes [13]. In an orthotopic PDAC model, the macsGESTALT system combined single‐cell transcriptomic and lineage information to demonstrate that hybrid EMT states possess greater invasive and metastatic potential and are associated with poorer prognosis [277]. In PNI, integrated scRNA‐seq, snRNA‐seq, spatial transcriptomics, and multiplex immunohistochemistry showed that NLRP3^+^ TAMs and myofibroblast‐like CAFs surround invaded nerves in highly neuroinvasive tissues, whereas TGFBI^+^ SCs accumulate at the invasive front and promote pancreatic cancer cell migration and invasion [278]. In addition, 2D/3D spatial multiomics models that integrate copy number inference, somatic mutation mapping, single‐nucleus transcriptomics, and CODEX protein imaging can reconstruct ecological evolutionary models within tissue architecture. These approaches identify tumor‐immune interfaces that cannot be adequately captured by conventional 2D sections alone and are valuable for understanding metastatic heterogeneity, immune exclusion, and therapy resistance [279]. Importantly, single‐cell and spatial approaches can identify candidate cell states, spatial colocalization patterns, and putative communication axes, but cannot independently establish causality. Integrating patient specimens, spatial multiomics, organoid and chip‐based models, animal functional validation, and lineage tracing is therefore essential for robust metastatic stratification and the development of mechanism guided therapeutic interventions.

## 7 Advances and Translational Challenges in Targeting Metastasis

The goal of antimetastatic therapy should extend beyond reducing established tumor burden to targeting critical bottlenecks across the metastatic cascade. These strategies may target cancer cell plasticity, reduce MTCs and MTC clusters, vascular and stromal barriers, immune exclusion, neural–tumor communication, dormant MTCs, and microbiome‐mediated regulation. The translational value of each strategy depends on its alignment with tumor type, metastatic stage, and metastasis biomarker profiles. Current clinical trials targeting metastatic processes are summarized in Table 3.

**TABLE 3:** Representative clinical trials targeting the metastatic cascade and migratory tumor microenvironment.

Clinical trial	Phase	Drug/intervention	Target	Enrollment	Combined therapy	Active comparator	Cancer type	Objectives	Current status	Primary outcomes	References
NCT03631706	III	Bintrafusp alfa (M7824)	PD‐L1 and TGF‐β	304	∖	Pembrolizumab	NSCLC	Evaluate the efficacy and safety of bintrafusp alfa (M7824) compared with pembrolizumab in participants with advanced NSCLC with high tumor PD‐L1 expression, with no EGFR mutation or ALK translocation.	Completed	PFS, OS	[280]
NCT03833661	II	Bintrafusp alfa (M7824)	PD‐L1 and TGF‐β	159	∖	∖	Metastatic biliary tract cancer	Evaluate M7824 monotherapy in participants with advanced or metastatic biliary tract cancer who failed or were intolerant to first‐line (1L) chemotherapy.	Completed	Confirmed objective response	[281]
NCT03724851	Ib/IIa	Vactosertib (TEW‐7197)	ALK5	120	Pembrolizumab	∖	Metastatic colorectal and gastric cancer	Assess safety, tolerability, pharmacokinetics, and antitumor activity of vactosertib in combination with pembrolizumab in patients with metastatic CRC including CMS4 or diffuse GC/GEJC with two phases.	Completed	Maximum tolerated dose, safety and tolerability	[282]
NCT03184571	II	Bemcentinib	Axl	99	Pembrolizumab	∖	Lung adenocarcinoma	Assess the antitumor activity and safety of bemcentinib in combination with pembrolizumab in patients with previously treated, advanced lung adenocarcinoma.	Completed	ORR	[283]
NCT02546531	I	Defactinib (VS‐6063)	FAK	43	Pembrolizumab, gemcitabine	∖	Pancreatic cancer	Evaluate the effect of FAK inhibitor in combination with a PD‐1 antagonist and chemotherapy in patients with pancreatic cancer.	Completed	RP2D	[284]
NCT03875820	I	Defactinib (VS‐6063)	FAK	87	VS‐6766 (RO5126766)	∖	NSCLC, pancreatic cancer, CRC, low‐grade serous ovarian cancer, endometrioid cancer	Determine the MTD and RP2D, and characterize the safety and tolerability and to assess the pharmacodynamic activity of the combination.	Active, not recruiting	RP2D, adverse events	[285]
NCT03928210	I	Digoxin	CTC clusters	58	∖	∖	Metastatic breast cancer	Investigate whether cardiac glycosides are able to disrupt CTC clusters in breast cancer patients.	Completed	Change in mean CTC cluster size	[286]
NCT02647099	III	Aspirin	Platelet	626	∖	Placebo	CRC	Evaluate the survival effect of aspirin in patient colorectal cancer and somatic mutations in PI3K pathway.	Active, not recruiting	Time to recurrence	[287]
NCT02826486	II	BL‐8040	CXCR4	80	Pembrolizumab	BL‐8040/Pembrolizumab in combination with liposomal irinotecan/5‐FU/LV	Pancreatic adenocarcinoma	Assess the efficacy and safety of BL‐8040 in combination with pembrolizumab and BL‐8040/ Pembrolizumab in combination with liposomal irinotecan/5‐fluorouracil/leucovorin in patients with metastatic pancreatic adenocarcinoma.	Completed	ORR	[288]
NCT03384836	I/II	Propranolol hydrochloride	β‐Adrenergic receptors	47	Pembrolizumab	∖	Melanoma	Study the side effects and best dose of propranolol hydrochloride in combination with pembrolizumab in patients with unresectable Stage IIIC–IV melanoma.	Suspended	DLT, ORR	^289^
NCT01472198	II	Simtuzumab	LOXL2	250	Gemcitabine	Placebo	Pancreatic cancer	Compare the efficacy of simtuzumab (GS‐6624) versus placebo in combination with gemcitabine in adults with pancreatic cancer.	Completed	PFS	[289]
NCT02777710	I	Pexidartinib	CSF‐1R	48	Durvalumab	∖	Metastatic colorectal or pancreatic cancer	Evaluate the safety and clinical activity of a combination of the anti‐CSF1R agent pexidartinib and the anti‐PD‐L1 agent durvalumab in patients with advanced/metastatic colorectal or pancreatic cancer.	Completed	DLT, ORR	[290]
NCT03032406	II	Hydroxychloroquine	Autophagy	53	Everolimus	Everolimus alone or hydroxychloroquine alone	Breast cancer	Evaluate the feasibility of administering hydroxychloroquine, everolimus or the combination in patients who have completed primary therapy for breast cancer and harbor bone marrow disseminated tumor cells.	Active, not recruiting	Number of participants who completed 6 cycles of protocol treatment without Grade 3 or 4 toxicity	[291]
NCT03341143	II	Fecal microbiota transplant	Microbiome	20	Pembrolizumab	∖	PD‐1 resistant/refractory melanoma	Determine whether FMT can enhance therapeutic efficacy of melanoma.	Completed	ORR	[292]
NCT03772899	I	Fecal microbiota transplantation	Microbiome	20	Pembrolizumab or nivolumab	∖	Melanoma	Examine the safety of combining fecal microbiota transplantation with pembrolizumab or nivolumab in melanoma patients.	Active, not recruiting	Safety	[293]
NCT03829111	I	Clostridium butyricum CBM 588 probiotic strain	Microbiome	30	Ipilimumab and nivolumab	Ipilimumab in combination with nivolumab	Metastatic kidney cancer	Study the effect of CBM588 in combination with nivolumab and ipilimumab in treating patients with Stage IV metastatic kidney cancer.	Completed	Change in bifidobacterium composition of stool	[294]
NCT05122546	I	Clostridium butyricum CBM 588 probiotic strain	Microbiome	31	Cabozantinib S‐malate and nivolumab	Cabozantinib S‐malate in combination with nivolumab	Metastatic kidney cancer	Evaluate the effects of CBM588 in combination with standard therapies, nivolumab and cabozantinib, in treating patients with advanced/metastatic kidney cancer.	Active, not recruiting	Change in bifidobacterium composition of stool	[295]
NCT04758507	I/II	Fecal microbial transplantation	Microbiome	50	∖	Placebo FMT	Advanced renal cell carcinoma	Evaluate the efficacy of targeted FMT in improving response rates to ICIs in subjects with advanced renal cell carcinoma.	Completed	Number of participants free from tumor progression	[296]
NCT04523857	II	Hydroxychloroquine	Autophagy	44	Abemaciclib	Abemaciclib alone	Breast cancer	Test whether hydroxychloroquine with or without abemaciclib can reduce or eliminate disseminated tumor cells in bone marrow.	Recruiting	Incidence of adverse events, change in bone marrow DTC number	∖
NCT02907099	II	BL‐8040	CXCR4	20	Pembrolizumab	∖	Metastatic pancreatic cancer	To assess the overall response rate after treatment with CXCR4 antagonist BL‐8040 and pembrolizumab.	Completed	ORR	∖
NCT03373188	I	VX15/2503	SEMA4D	10	Nivolumab, ipilimumab	∖	Pancreatic cancer, CRC	Evaluate the effect of anti‐SEMA4D monoclonal antibody in combination with immune checkpoint inhibitors in the TME and peripheral blood.	Completed	Treatment effects on tumor CD8+ T cell infiltration	∖
NCT06331169	Ib	Anlotinib	VEGFR, FGFR, PDGFR	42	Trastuzumab, deruxtecan	∖	Metastatic breast cancer	Evaluate the safety, tolerability, and efficacy of anlotinib and trastuzumab deruxtecan in HER2‐low unresectable and/or metastatic breast cancer.	Recruiting	ORR, RP2D	∖
NCT02452008	II	Galunisertib (LY2157299)	TGF‐β RI	60	Enzalutamide	Enzalutamide alone	Metastatic castration‐resistant prostate cancer	Compare the PFS of patients with metastatic castration‐resistant prostate cancer treated with enzalutamide in combination with LY2157299 versus enzalutamide alone.	Active, not recruiting	PFS	∖
NCT04599140	Ib/II	SX‐682	CXCR1/2	51	Nivolumab	∖	Metastatic CRC	Study the side effects and best dose of SX‐682 that can be given alone and in combination with nivolumab in treating patients with RAS‐mutated, microsatellite stable metastatic or unresectable CRC.	Active, not recruiting	Incidence of adverse events	∖
NCT06228053	II	SX‐682	CXCR1/2	53	Enzalutamide	∖	Metastatic prostate cancer	Study the combination of SX‐682 plus enzalutamide in men with metastatic castration‐resistant prostate cancer.	Recruiting	Clinical Benefit	∖
NCT05200559	I/II	E7777	Tregs	70	Pembrolizumab	∖	Ovarian cancer	Explore the effect of targeting tumor‐associated Tregs with E7777 combined with pembrolizumab in ovarian cancer patients.	Active, not recruiting	RP2D, changes of Tregs, CD8+ T cells, myeloid cells in tumor and peripheral blood.	∖
NCT05774899	I/II	CB‐103	NOTCH pathway	32	Abemaciclib, lenvatinib	∖	Adenoid cystic carcinoma	Evaluate the survival outcomes and effects on tumor growth of NOTCH signaling inhibition in combination with CDK4/6 inhibitor and VEGFR‐TKI in patients with NOTCH active advanced ACC.	Recruiting	PFS	∖
NCT03839498	II	Axitinib	VEGFR1/2/3	6	∖	∖	Pheochromocytoma/paraganglioma	Determine the response rate of metastatic or locally advanced pheochromocytoma/paraganglioma to axitinib.	Active, not recruiting	Response rate	∖
NCT07008703	I/II	SSS40	NGF	132	∖	∖	Bone metastasis	Evaluating the safety, tolerability, and preliminary efficacy of NGF antibody injection in patients with bone metastasis cancer pain.	Recruiting	Incidence and severity of adverse events and serious adverse events	∖
NCT04563338	II	Atezolizumab	PD‐L1	36	Bevacizumab	Atezolizumab alone	Metastatic HCC and NSCLC	Evaluate the effect of atezolizumab, with or without bevacizumab, in patients with inoperable HCC and NSCLC liver metastasis.	Active, not recruiting	PFS	∖

*Data source*: ClinicalTrials.gov website (https://clinicaltrials.gov/).

Abbreviations: 5‐FU/LV, 5‐fluorouracil/leucovorin; ACC, adenoid cystic carcinoma; ALK5, activin receptor‐like kinase‐5; CDK4/6, cyclin‐dependent kinase 4/6; CRC, colorectal cancer; CSF‐1R, colony‐stimulating factor‐1 receptor; CTC, circulating tumor cells; CXCR1/2, C–X–C chemokine receptor 1/2; CXCR4, C–X–C chemokine receptor 4; DLT, dose limiting toxicities; DTC, disseminated tumor cell; EGFR, epidermal growth factor receptor; FAK, focal adhesion kinase; FGFR, fibroblast growth factor receptor; FMT, fecal microbiota transplantation; GC/GEJC, gastric cancer or gastroesophageal junction carcinoma; HCC, hepatocellular carcinoma; LOXL2, lysyl oxidase‐like protein 2; MTD, maximum tolerated dose; NGF, nerve growth factor; NSCLC, non‐small cell lung cancer; ORR, objective response rate; OS, overall survival; PD‐1, programmed cell death protein 1; PDGF, platelet‐derived growth factor; PDGFR, platelet‐derived growth factor receptor; PD‐L1, programmed cell death 1 ligand 1; PFS, progression‐free survival; RP2D, recommended Phase Ⅱ dose; SEMA4D, semaphorin 4D; TGF‐β, transforming growth factor‐β; TKI, tyrosine kinase inhibitor; TME, tumor microenvironment; Tregs, regulatory T cells; VEGFR, vascular endothelial growth factor receptor.

### 7.1 Liquid Biopsy: From Metastatic Risk Identification to Treatment Stratification

MTCs and circulating tumor DNA (ctDNA) are currently the most clinically accessible liquid biopsy biomarkers associated with metastasis. Elevated MTC counts are strongly associated with poor prognosis across multiple cancers [297]. For instance, postoperative quantification of small triploid EpCAM‐MTCs (≥5), EpCAM^+^ pentaploid circulating tumor stem cells (≥1), and CTM (≥1) independently predicts early HCC relapse and poor disease‐free survival, enabling risk‐adapted postoperative surveillance [298]. Similarly, postoperative MTC counts exceeding 3 cells per 4 mL of peripheral blood reveals significant higher risk of recurrence and metastasis [299]. Early‐stage liquid biopsy assays that integrate MTC size, surface antigens, and phenotypic characteristics have demonstrated high sensitivity and specificity for cancer detection [300, 301].

Besides CTCs, ctDNA provides a valuable indicator of metastatic risk and tumor progression. In metastatic castration‐resistant prostate cancer, a high baseline ctDNA fraction exhibit enhanced potential for liver metastasis and a shortened time to progression after initiation of androgen deprivation therapy [302]. In lung adenocarcinoma, preoperative ctDNA negativity correlates with significantly improved OS and prolonged freedom from relapse [303]. Moreover, ctDNA methylation profiles have diagnostic value in CRC, and the methylation marker cg10673833 provides prognostic information beyond standard clinical characteristics [304]. Accordingly, liquid biopsy can identify patients at high risk of recurrence, enable longitudinal monitoring of minimal residual disease, and provide a basis for metastasis prevention.

### 7.2 Targeting the Circulatory Phase: EMT and MTC Clusters

TGF‐β is a central inducer of EMT and a key link among cancer cell plasticity, CAF activation, immune exclusion, and metastasis. It is therefore an important but highly complex therapeutic target. Bintrafusp alfa, a bifunctional agent combining PD‐L1 blockade with TGF‐β trapping, did not outperform pembrolizumab in a Phase III trial of PD‐L1 high advanced NSCLC and was discontinued after failing to meet its primary endpoint [280]. In advanced biliary tract cancer, bintrafusp alfa achieved only a limited ORR and short PFS [281]. These results suggest that broad TGF‐β inhibition, despite its strong mechanistic rationale, may have a restricted therapeutic window because of its roles in tissue homeostasis, pleiotropic immune regulation, and the heterogeneity of advanced metastatic lesions. More selective inhibition of TGF‐β signaling, or targeting EMT‐associated pathways in combination with immunotherapy, has nevertheless shown preliminary signals of activity. Early studies of vactosertib plus pembrolizumab and bemcentinib plus pembrolizumab suggest that selected patients may benefit, but their clinical value requires confirmation in randomized trials with biomarker‐based selection according to TGF‐β activation and immune exclusion phenotypes [282, 283]. In addition, MTC clusters represent one of the few metastatic process targets that has entered clinical intervention studies. Digoxin, a Na^+^/K^+^‐ATPase inhibitor, reduces intercellular adhesion and cell cycle‐associated transcriptional programs. In a study in metastatic breast cancer, digoxin reduced MTC cluster size by an average of 2.2 MTCs per cluster, providing early clinical evidence that MTC clusters may be therapeutically actionable rather than merely prognostic markers [286].

### 7.3 Targeting the Metastatic Niche: ECM, Adhesion, and Immune Responses

Stromal remodeling is a key driver of invasion, vascular abnormalities, and immune exclusion. However, inhibition of a single stromal target is often insufficient to reverse an established metastatic microenvironment. For instance, in preclinical PDAC models, LOXL2 inhibition reduced fibroblast activation, ECM deposition, angiogenesis, and metastasis. However, in a randomized Phase II trial, targeting LOXL2 by combining simtuzumab with gemcitabine did not improve the survival of metastatic PDAC, indicating that isolated stromal inhibition is unlikely to reverse a mature metastatic niche [289]. FAK inhibition may offer broader effects by simultaneously suppressing integrin‐mediated adhesion, EMT, cancer cell migration, and TAM/Treg infiltration. In a study involving 43 cases of refractory PDAC patients, FAK inhibitor defactinib combined with pembrolizumab and gemcitabine achieved an 80% disease control rate, but the ORR was only 5% and median PFS was 3.6 months. Although paired biopsies showed reduced p‐FAK, TAMs, and Tregs, together with increased CD8^+^ T cell infiltration, these changes did not translate into clear clinical benefit [284]. In another low‐grade serous ovarian cancer cohort of the FRAME study, defactinib plus avutometinib achieved an ORR of 42.3% and a median PFS of 20.1 months. In contrast, no objective responses were observed in PDAC or CRC cohorts, further demonstrating that the efficacy of stromal targeting may depend on tumor type, molecular subtype, and the therapeutic backbone [285].

Targeting immune regulation represents another major therapeutic direction. STK11/LKB1‐mutant NSCLC lacks the TCF1^+^PD‐1^+^CD8^+^ T cell population associated with response to PD‐1 blockade. Preclinical studies show that AXL inhibition relieves suppression of Type I interferon signaling in dendritic cells, promotes expansion of TCF1^+^PD‐1^+^CD8^+^ T cells through IFNAR, and synergizes with PD‐1 blockade. In immunocompetent and humanized mouse models, the AXL inhibitor bemcentinib combined with PD‐1 blockade markedly controlled STK11/LKB1‐deficient tumors. An ongoing Phase II study, however, has reported only a limited number of durable partial responses and cases of stable disease [283]. In the COMBAT trial, the CXCR4 antagonist motixafortide mobilized CXCR4^+^ T cells into the peripheral circulation, reduced circulating Tregs, and increased intratumoral CD8^+^GZMB^+^ cells. Motixafortide plus pembrolizumab nevertheless achieved an ORR of only 3.4%. Addition of liposomal irinotecan and 5‐fluorouracil increased the ORR and disease‐control rate to 32 and 77%, respectively. CXCR4 blockade may therefore be more effective as a sensitizer to chemotherapy and immunotherapy [288]. Multitarget intervention in the immune microenvironment can also fail because of pathway pleiotropy and treatment‐related toxicity. For example, bintrafusp alfa simultaneously targets PD‐L1 and TGF‐β‐mediated T cell exclusion, but achieved an ORR of only 10.7% and median PFS of 1.8 months in biliary tract cancer. In a Phase III study, it failed to improve PFS, overall survival, or ORR over first‐line pembrolizumab and caused more treatment‐related adverse events [280, 281].

Targeting dormant cancer cells is a promising strategy for metastasis prevention. Preclinical work underlying the CLEVER study showed that dormant breast cancer cells have low mTOR activity and a strong dependence on autophagy; inhibition of either mTOR or autophagy reduced residual cancer cells and delayed recurrence in mice. In a Phase II study of 53 patients, everolimus, hydroxychloroquine, and their combination each increased clearance of MTCs from bone marrow. The combination showed no clear advantage over either monotherapy, however, and the small number of recurrences precluded assessment of a survival benefit [291]. Translation in this field remains early and requires trial designs distinct from those used in advanced solid tumors, including highly sensitive minimal residual disease assays, extended follow‐up, and recurrence‐based primary endpoints. More broadly, successful targeting of the metastatic microenvironment depends not on simply adding more targets, but on using spatial pharmacodynamics and molecular biomarkers to establish which barrier has been disrupted, which cells actually participate in dissemination or enter the lesion, and whether the intervention improves clinically meaningful survival.

### 7.4 Targeting Neural Communication

Neural regulation has recently emerged as an antimetastatic target, but its effects are strongly dependent on cancer type and receptor pathway. In murine PDAC, cancer cell ANXA2 promotes SEMA3D secretion, which enhances PLXND1‐dependent MTC migration and acts on PLXND1 in sensory nerves to stimulate neurite outgrowth and intratumoral neural remodeling. SEMA3D knockdown or sensory neuron‐specific PlxnD1 loss reduces neural density and distant metastasis without substantially altering primary tumor volume, indicating that the perineural niche can independently regulate metastatic competence [67]. Neurogenic factors such as NGF are also promising neuroimmune targets. In mouse models, pharmacological blockade of NGF–TRK signaling with larotrectinib, combined with adoptive T cell transfer, IL‐2, and PD‐1 blockade, increased complete responses and protection against tumor rechallenge [305].

Clinically, β‐adrenergic signaling expands MDSCs and suppresses CD8^+^ T cells and NK cells, making it an important mediator of systemic immune dysfunction. In an early study of nine patients with advanced melanoma, the β‐blocker propranolol combined with pembrolizumab showed acceptable safety and an ORR of 78%; the small, uncontrolled cohort, however, precludes firm conclusions regarding efficacy [306]. Another study showed that short‐course perioperative propranolol plus etodolac reduced metastatic biomarkers, including IL‐6, C‐reactive protein, STAT3, and mesenchymal transcriptional programs, while preserving IL‐12, IFN‐γ, and NK cell‐associated functions. Although this regimen concurrently inhibited neural stress and inflammatory pathways, the findings mainly demonstrate immune pharmacodynamic effects and do not establish a reduction in distant recurrence [307]. Vagal signaling through the M3 receptor promotes gastric cancer growth and metabolic adaptation. Vagotomy may be associated with improved survival in locally unresectable gastric cancer, but has shown no clear benefit after distant metastasis [308]. By contrast, vagal–CHRM1 cholinergic signaling suppresses EGFR/MAPK and PI3K/AKT, reduces pancreatic CSCs, and restrains liver metastatic growth; vagotomy or Chrm1 loss accelerates liver metastasis, whereas the muscarinic agonist bethanechol prolongs survival in KPC mice [309]. Neural‐targeted therapy should therefore address specific ligand–receptor axes and disease stages rather than rely on nonselective denervation.

### 7.5 Targeting the Microbiome

Microbiome intervention provides an alternative route for systemic remodeling of immune and metabolic niches. Fecal microbiota transplantation (FMT) may enhance responses to immune checkpoint inhibitors in selected metastatic tumors. In advanced melanoma, FMT combined with pembrolizumab or nivolumab was associated with sustained donor‐strain engraftment in responders, enhanced antigen presentation in the gut and tumor, increased CD8^+^ T cell activation, and reduced myeloid suppression. Meanwhile, objective responses were observed in a subset of studied patients, supporting the feasibility and acceptable safety of this approach [292, 293, 310]. However, most FMT studies remain small, single‐arm investigations and cannot attribute survival benefit definitively to microbiome modulation. In the Phase IIa TACITO trial, metastatic renal cell carcinoma patients receiving FMT in addition to standard first‐line pembrolizumab plus axitinib showed increases in median survival and gut microbial diversity. Nevertheless, the primary endpoint of 12‐month PFS did not reach statistical significance [292, 294, 295, 296]. FMT should therefore currently be viewed as an exploratory immunotherapy sensitizing strategy. Future studies need to define optimal donor‐recipient matching, stability of microbial engraftment, combination treatment strategies, and long term infectious risks.

### 7.6 Challenges in Clinical Translation

No broadly reproducible, pan‐cancer antimetastatic treatment paradigm is currently available, reflecting persistent mismatches among therapeutic targets, metastatic stage, microenvironmental context, and clinical endpoints. Despite substantial recent progress, the field still lacks a metastasis‐specific and widely accepted therapeutic framework. Several challenges impede translation. First, metastasis is highly cancer type, time, and space dependent. Dormant disease and micrometastases are difficult to detect early, whereas advanced multisite lesions can differ markedly from one another and generate both local and systemic treatment resistance. Second, tumor‐microenvironment interactions vary across habitats, and no dominant mechanism or molecular target has been universally established. Third, clinically validated tools and biomarkers for detecting metastasis remain inadequate. Finally, most early studies are limited by small sample sizes, nonrandomized designs, and lack of metastasis‐specific endpoints, making true treatment activity difficult to distinguish from selection bias and variation in natural history.

Future trials should move from empirical combinations toward mechanism matched, biomarker stratified designs. In the adjuvant or minimal residual disease setting, priority should be given to interventions that suppress MTC survival and intravascular protection, PMN formation, and reactivation of dormant MTCs. Clearance of ctDNA or MTCs, metastasis‐free survival, and time to recurrence should serve as key endpoints. In patients with established organ metastases, combination strategies should be selected according to the organ‐specific metastatic microenvironment and spatial immune state, with survival outcomes used for confirmatory evaluation. Only by aligning treatment timing, metastatic state, predictive biomarkers, and clinically meaningful endpoints can metastasis‐directed therapy produce durable clinical benefit.

## 8 Conclusion and Future Prospects

In recent decades, technological innovations and theoretical breakthroughs have driven substantial progress in tumor metastasis research. The prevailing view now holds that cancer cell metastasis is not the result of a simple linear accumulation of genetic alterations in malignant cells, but rather it is a spatiotemporal dynamic process jointly driven by intrinsic cancer cell evolution and continuous microenvironmental selection. Researchers have systematically dissected the complex evolutionary mechanisms of cancer cells, with findings emphasizing that multidimensional interactions with the TME are core determinants governing this spatiotemporal dynamic process. The continuous evolution of MTCs during metastasis results from both active phenotypic remodeling and passive adaptation under the selective pressures of local niches, ultimately enabling cancer cells to acquire metastasis stage‐specific capabilities in migration/ECM remodeling, metabolic adaptation, and immune evasion. Primary sites, migratory niches, and metastatic organs—as the contexts shaping MTC dissemination—exhibit rich heterogeneity in physicochemical properties, cellular composition, and signaling networks, while the complex interactions between MTCs and local niches collectively determine the final metastatic outcome. Notably, during the window period from departure from the primary site to eventual metastatic colonization, MTCs across cancer types exist in an extremely vulnerable state and navigate through three major dissemination routes: hematogenous, lymphatic, and perineural. In this review, we proposed the concept of the mTME and systematically described the complex, spatiotemporally dependent interaction between MTCs and their local niches across distinct dissemination routes. We also outline the most urgent technical and translational challenges currently facing tumor metastasis research.

The spatiotemporal dynamics of tumor metastasis provide multiple potential windows for therapeutic intervention, and numerous preclinical and clinical studies have identified and developed a range of promising molecular targets and pathways. However, we argue that the development of antimetastatic interventions must urgently shift from target‐centric empiricism toward precision strategies that match timing, routes, biomarkers, and endpoints. In high‐risk localized disease or minimal residual disease stages, strategies such as disrupting MTC clusters, modulating the tumor immune niche, and suppressing dormant cell survival or reactivation may be more effective. Corresponding studies should use ctDNA clearance rate, CTC dynamics, progression‐free survival, metastasis‐free survival, and time to recurrence as appropriate endpoints. For established metastatic disease, treatment selection should be more heavily guided by organ‐specific vascular, stromal, metabolic, and immunological features, and is more likely to achieve clinical benefit through rational combination strategies rather than isolated pathway inhibitors. Ultimately, the mTME provides a conceptual and translational platform for intervention prior to stable metastatic colonization, and researchers may successfully identify and validate shared vulnerable targets across cancer types within this framework. Nevertheless, the realization of clinical value depends on both the reproducible measurement of route‐specific vulnerabilities and whether they can be safely targeted, ultimately translating into sustained reductions in metastatic recurrence and cancer mortality.

To address these gaps, a clear experimental roadmap is urgently needed. Prospective longitudinal cohorts should integrate serial ctDNA analysis, phenotypically preserved MTC and MTC‐cluster profiling, circulating immune and platelet‐related markers, and paired primary, LN, and metastatic samples. These clinical datasets should be linked with spatial multiomics and lineage‐resolved analyses to identify route‐specific cell states and their interaction networks. Candidate mechanisms should be validated in stage‐matched experimental systems, including: perfused patient‐derived organoid‐on‐chip platforms incorporating reconstituted local niche stress components; complementary immunocompetent, humanized, and orthotopic in vivo models to assess the complete metastatic cascade; and inducible genetic perturbation or lineage‐tracing approaches to determine the temporal necessity of candidate pathways. Immediate technical challenges include preserving the native composition of rare MTC clusters during isolation, obtaining representative lymphatic and perineural samples, recapitulating dynamic mechanical and immune conditions in vitro, and standardizing measurements across platforms. Critically, multiomic discoveries must be integrated with functional perturbations and clinically relevant endpoints before candidate interactions can be considered therapeutically actionable.

## Author Contributions

JC. G. and JZ. L. contributed to the conception and design of this review. YP. Z. and WC. X. wrote the original draft. YP. Z. created the figures and tables. YB. W., HR. H., and Y. S. collected related literature, edited, and revised the manuscript. JZ. L., L. Z., and JC. G. conceived and supervised this project. YP. Z. and WC. X. contributed equally to this draft. All authors have read and approved this manuscript.

## Funding

This study was supported by National Natural Science Foundation of China (825B2083; 82573268), the National High Level Hospital Clinical Research Funding (2025‐PUMCH‐H‐011), the Fundamental Research Funds for the Central Universities, Peking Union Medical College (3332025119), and the Noncommunicable Chronic Diseases‐National Science and Technology Major Project (2025ZD0552415; 2025ZD0552400).

## Ethics Statement

The authors have nothing to report.

## Conflicts of Interest

The authors declare no conflicts of interest.

## Data Availability

The authors have nothing to report.
